# Multi-session CBM-I for social anxiety: examining psychopathology, cognitive, neural, and psychophysiological effects in a randomized controlled trial

**DOI:** 10.1038/s41398-026-04122-2

**Published:** 2026-05-22

**Authors:** Elinor Abado, Marius Kunna, Felix Würtz, Lilith Laflör, Laura Blank, Simon E. Blackwell, Elske Salemink, Dirk Adolph, Fanny Alexandra Dietel, Oliver T. Wolf, Jürgen Margraf, Marcella L. Woud

**Affiliations:** 1 Department of Clinical Psychology and Experimental Psychopathology, Georg-Elias-Müller-Institute of Psychology, University of Göttingen, Göttignen, Germany; 2 Mental Health Research and Treatment Center, Faculty of Psychology, Ruhr University Bochum, Bochum, Germany; 3 Department of Clinical Psychology, University of Utrecht, Utrecht, Netherlands; 4 Department of Clinical Psychology and Psychotherapy, University of Osnabrück, Osnabrück, Germany; 5 Department of Cognitive Psychology, Institute of Cognitive Neuroscience, Faculty of Psychology, Ruhr University Bochum, Bochum, Germany

**Keywords:** Human behaviour, Physiology

## Abstract

Cognitive Bias Modification – Interpretation (CBM-I) aims to alter maladaptive interpretations in social anxiety, yet effects are often small and outcome measures are diverse. Although CBM-I has shown promise, its underlying mechanisms remain unclear and integration with psychophysiological and neural measures has been limited. In this randomized controlled trial, eighty-eight participants with high levels of social anxiety completed two lab sessions, an online training in between, and online follow-up. Participants filled out questionnaires, completed interpretation bias tasks, and underwent neuro-psychophysiological assessments. Active CBM-I trained positive resolutions of ambiguous social scenarios, while the sham version used neutral scenarios. The primary outcome, i.e., scores on the Liebowitz Social Anxiety Scale (LSAS), decreased across time in both groups, without group differences. However, the Brief Fear of Negative Evaluation decreased only in the active group. Interpretation bias shifted more strongly toward positive outcomes in the active group. Autonomic measures confirmed sensitivity to stress induction but did not differentiate between conditions. Electrophysiological results paralleled subjective ratings, as participants exhibited ambivalent responses to socially relevant stimuli but clearly differentiated responses toward neutral stimuli. Baseline correlations indicated strong convergence across self-report and interpretation tasks. Mediation analyses showed that reductions in negative interpretations mediated the effect of the training group on LSAS scores at follow-up. These findings identify interpretation bias as a modifiable mechanism underlying social anxiety and underscore its role as a transdiagnostic marker. Targeting interpretation bias through easily accessible and applicable online interventions may strengthen preventive and therapeutic approaches for social anxiety and related disorders.

## Introduction

Social anxiety disorder (SAD) is a severe mental disorder characterized by persistent fear and avoidance of social situations due to concerns about negative evaluation [1]. With a lifetime prevalence of 6.7% [2], SAD is common and often chronic if untreated. Effective treatments, including Cognitive Behavioural Therapy (CBT) and pharmacological approaches, are well established [3, 4]. However, up to 50% of patients show insufficient response and remain symptomatic or relapse after treatment.

Interpretation bias (IB)—the tendency to resolve ambiguity in a preferential manner [5, 6]—plays a key role in maintaining social anxiety disorder (SAD) [7–9]. Individuals with SAD show a pronounced negative and reduced positive IB, e.g., interpreting ambiguous events (e.g., being called into a boss’s office) as socially threatening [10, 11]. IBs, in turn, evoke fear and maladaptive behaviours such as avoidance, which maintain anxiety by preventing corrective experiences [7, 8]. Consequently, modifying IB is crucial for reducing SAD symptoms. Cognitive Bias Modification - Interpretation (CBM-I) targets IB by repeatedly practicing the adaptive resolution of ambiguous, disorder-relevant scenarios [12, 13]. CBM-I has been used to both test the causal role of IB and as an intervention, with meta-analyses showing reductions in anxiety symptoms compared to control conditions [14].

CBM-I is similar to CBT in that both aim to reduce IB and to promote a shift toward more adaptive interpretations. In CBT, patients typically engage in a retrospective process of identifying negative interpretations, evaluating their consequences, and generating adaptive alternatives, all collaboratively with a therapist. In contrast, CBM-I uses computerized multi-trial training to repeatedly target a broader range of maladaptive interpretations, encouraging a gradual shift through repeated practice. There is some initial evidence that CBM has potential as a standalone intervention [15], but it also has clear value as an adjunct to CBT, serving as a mechanism-oriented tool to further enhance cognitive change [16]. Studies comparing CBT with CBM-I have found that although both conditions reduce IBs, CBM-I was more effective [17]. A study comparing CBT with CBM in adolescents reported comparable short-term reductions in social anxiety, whereas CBT produced stronger and more durable reductions in test anxiety [18]. Thus, CBM-I and CBT may offer mechanistically different yet complementary approaches.

Despite considerable progress in recent years, multimodal studies assessing the effects of CBM-I remain scarce. Event-related potentials (ERPs) provide a promising measure, offering insight into the temporal dynamics of IB, indexing expectancy violation [19]. Specifically, the N400 component—a marker of verbal expectancy violation [20, 21] — shows greater amplitudes when a target word is unexpected compared to when it is expected, thus reflecting interpretation. Few studies have examined IB as expressed in ERPs in social anxiety [19], with mixed results: one found no N400 expression of IB [22], while another found reduced positive and increased negative interpretations in social anxiety and depression, compared to a control group [23]. In the latter study, the same pattern was also reflected in behavioral measures (reaction time and accuracy) [23]. Thus, the N400 may indicate both positive and negative interpretations [21]. Complementary, frontal alpha asymmetry (FAA) at rest indexes affective styles, rather than event-related responses with right-lateralized FAA indexing negative or avoidant tendencies [24]. FAA has also been associated with attention bias to threat [25] and shown to normalize following CBT for social anxiety [26]. These results suggest that FAA may relate to cognitive processes and is sensitive to interventions. However, no studies have examined the relationship between FAA and IB. Together, N400 and FAA could provide a comprehensive view of neural responses related to CBM-I effects. While prior IB and CBM-I studies focused on symptoms and behavior, ERPs reveal the temporal unfolding of IB, and resting-state EEG can capture individual neural differences. Such an integrative approach is essential for refining theoretical models, clarifying mechanisms of change, and improving the interpretability of CBM-I effects.

Importantly, IB change may impact not only symptoms but also psychophysiological responses to stress, such as heart rate (HR), heart rate variability (HRV), salivary cortisol, and salivary alpha-amylase (sAA). Previous studies examined HR and HRV as measures of general hypervigilance and stress reactivity, but few have examined whether stress reactivity changes following CBM-I [19]. Notably, a previous study [27] found improved HR recovery after a stress task post-training, although self-report measures showed no improvement, highlighting the added value of physiological measures. As for cortisol, Turton et al. (2018) applied a single-session CBM-I training in women with anorexia nervosa [28]. Results showed no significant transfer effects of CBM-I to salivary cortisol levels after a stressor. However, as physiological responses are central to social anxiety, further research using diverse measures is needed.

Accordingly, the present multimodal, multisession, single-blind, randomized controlled trial (RCT) had two main aims: the first aim was to examine whether reduction in IB following CBM-I would lead to reductions in social anxiety symptoms at follow-up (primary outcome). This timeframe was selected because the follow-up assessment was considered the most appropriate time point for evaluating symptom change, i.e., it allowed participants sufficient time to consolidate and apply the learning before potential effects on symptoms could be observed. The second aim was to investigate CBM-I-related reductions in multifaceted measures of IB, psychopathology symptoms, neurophysiological indices, and stress reactivity. These secondary outcomes included self-report measures of social anxiety, anxiety sensitivity, anxiety, depression, and stress, measures of IB, neurophysiological indices (FAA and N400), psychophysiological measures (HR and HRV), and endocrine stress responses (sAA and cortisol). In the current RCT, individuals with elevated social anxiety completed six online CBM-I sessions or a sham training. All outcomes were assessed pre- and post-training, with social anxiety and IB measures additionally collected at a 1-week follow-up.

We hypothesised that, compared to participants allocated to the sham training control condition, participants allocated to the CBM-I condition would show greater reductions in social anxiety symptoms at follow-up, compared to baseline. We hypothesized that this reduction would also extend to IB, electrophysiological, and psychophysiological measures. Finally, we hypothesised that the effects of CBM-I vs. sham training on social anxiety symptoms at follow-up would be mediated via effects on IB at post-training.

## Methods

### Participants

Eligibility criteria included age between 18 and 65 and a score of ≥ 52 on the Social Phobia and Anxiety Inventory, German version (SPAI-G; 0–6 scale [29, 30]), indicating elevated levels of social anxiety [31] (see Supplementary Materials for all criteria). Participants were recruited via e.g., social media and received time-contingent reimbursement per assessment (up to 100 € or 10 h of course credits).

The study was conducted at the Mental Health Research and Treatment Centre of the Faculty of Psychology, Ruhr University Bochum (Germany) and approved by the local ethics committee (approval #582). Informed consent was obtained from all participants. The study was conducted in accordance with the CONSORT 2010 guidelines for RCTs [32].

The required sample size was estimated via an a priori power analysis using G*Power [33], based on the expected between-group difference for the primary outcome (change in LSAS from baseline to follow-up) Following a meta-analytic estimate of CBM-I vs. sham effects on social anxiety symptoms (*d* = 0.98 [0.31, 1.65] [14], we aimed for 80% power to detect a more conservative effect (*d* = 0.65) similar to previous studies [34]. A G*Power analysis using the independent t-test program indicated *n* = 39 per group would be sufficient. To account for attrition *N* = 88 (*n* = 44 per group) were randomized.

The RCT ran from June 2023 to May 2024. Recruitment stopped when the target sample size was reached. Participants were randomly assigned to either active or control sham group following completion of all baseline measures via a web-based interface (see Supplementary Materials for full details). Randomization was stratified by gender in combination with use of hormonal contraception, baseline score on the Liebowitz Social Anxiety Scale [35, 36] (< 75 vs. ≥ 75), and age (40 vs. ≥ 40). For the full list of demographic and personal data collected, please see the Supplementary Materials. There was no patient or public involvement in the study, as the focus was on examining mechanisms rather than evaluating efficacy of an intervention.

### Design

The study was a two-arm parallel-group randomised controlled superiority trial with a 1:1 allocation ratio. Both interventions were web-based. Overall, the study’s design includes 2 variables: *group* as a between-subject variable (CBM-I/sham), and *time* as a within-subjective variable (baseline, which took place pre-training, post-training (1-week post-baseline), and follow-up (2 weeks post-baseline); T1, T2, and T3, respectively).

### Procedure

**Fig. 1:**
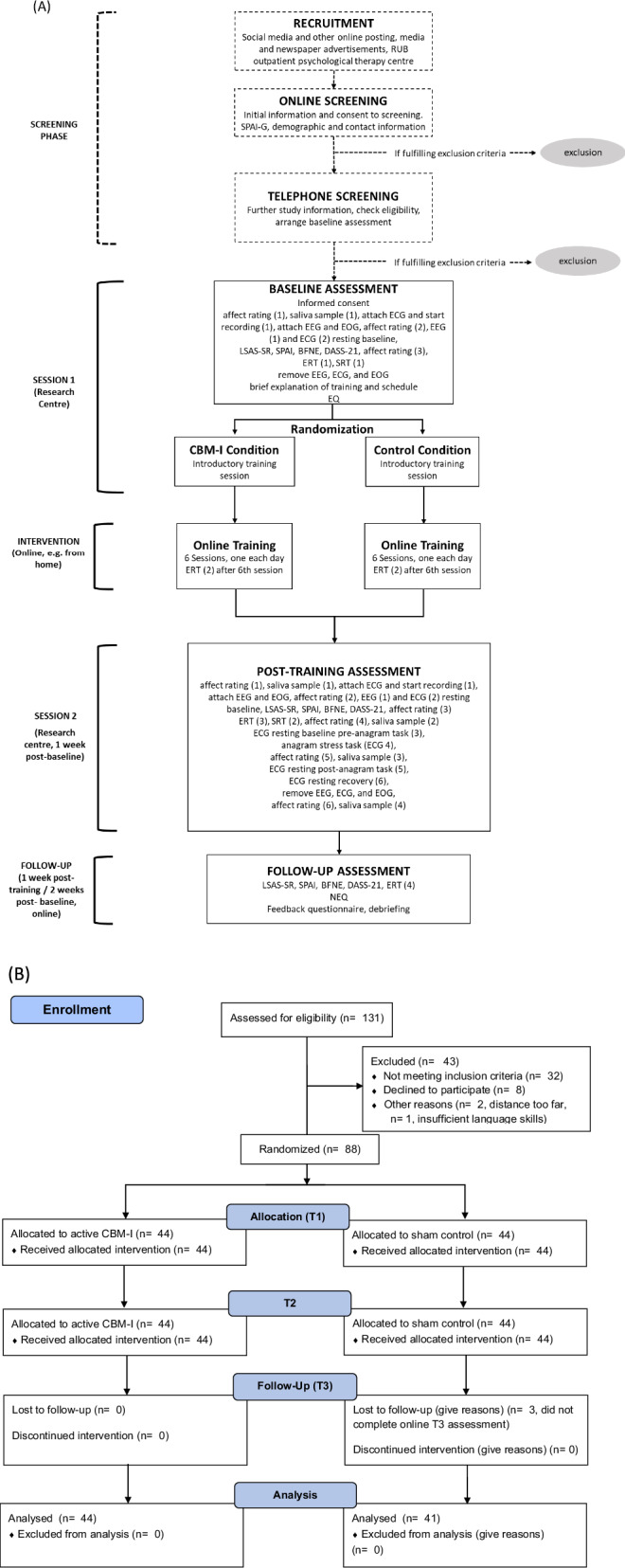
Study Procedure. **A** Flow chart detailing the study procedure. **B** CONSORT chart detailing participant screening and drop-out. There was no participant drop-out from T1 to T2.

The two multifaceted assessments (i.e., T1 and T2) were conducted face-to-face in the lab, the follow-up was conducted online (i.e., T3). For more details on the procedure and a CONSORT chart detailing participant screening and drop-out, see Fig. 1.

#### CBM-I

The CBM-I was presented as a sentence completion task, without reference to its training purpose. The training began with an introductory lab session, followed by six daily online training sessions, and was based on the Ambiguous Scenario Task-Training Version (AST [37, 38]). Each trial presented an ambiguous scenario resolved with a positive word fragment (e.g., “You are standing in a circle with your group of friends, and you are telling each other about your last weekend. When it’s your turn, you notice that your stories result in great laughter. You think that the others found your stories f_n_y [funny]”), which participants completed. In 25% of trials, comprehension questions followed. Each session included 45 trials in 5 blocks of 9 scenarios, presented in random order, with 100% positive resolutions. Positive endings were introduced gradually, starting mildly for the first three sessions and more intensely in the final three, to reduce rejection of overly positive outcomes [16, 39]. Sessions lasted ~15–20 min.

#### Sham training

To isolate CBM-I’s effects, a control condition was used. It matched CBM-I in format, time, and engagement but used neutral, non-social, ambiguous scenarios (e.g., “In the morning, you make your breakfast. You spread cream cheese on a slice of bread and make a cup of coffee. You pour the coffee into a cup and add some milk and sugar in the c_p [cup]”).

### Measures

#### Primary outcome

Liebowitz Social Anxiety Scale, Self-Report (LSAS-SR): LSAS-SR was used to measure symptoms of social anxiety within the last seven days [35, 40]. The questionnaire includes 24 different social situations (13 performance-related; 11 social interaction-related) and participants rate them in terms of fear and avoidance, using 4-point Likert scale of 0 (none/never) to 3 (severe/usually). A sum score of 30 and higher are indicative of social anxiety, and 60 and higher for generalised social anxiety disorder [41, 42]. The primary outcome measure was change in total score on the LSAS-SR from baseline (T1) to follow-up (T3).

#### Secondary outcomes

##### Symptom measures

All symptom measures were administered at all three timepoints (T1, T2, and T3). Unless stated otherwise, higher total scores on these scales indicate higher symptom severity.

Social Phobia and Anxiety Inventory, German version (SPAI-G): The 22-item SPAI-G [29, 30] was used to screen for social anxiety and as a secondary outcome measure. This brief version of the original SPAI contains the social phobia subscale encompassing cognitive, somatic, and emotional reactions to fear-provoking situations, rated on a 7-point Likert scale (0 = never to 6 = always).

Brief Fear of Negative Evaluation Scale (BFNE): The 12-item Fear of Negative Evaluation scale (BFNE [43, 44]) was used to assess fear of being negatively evaluated by others in social situations. Items are rated on a Likert scale (1 = not at all characteristic of me to 5 = extremely characteristic of me).

Depression, Anxiety, and Stress Scale–21 (DASS-21): Symptoms of depression, anxiety, and stress were assessed using the DASS-21 [45, 46], consisting of seven symptom-related items per subscale. Participants are instructed to rate the strength of each symptom in the past week using a 4-point Likert rating (0 = never to 3 = always).

##### IB measures

Each measure included two valence scores: one for positive IB and one for negative IB.

Encoding Recognition Task (ERT): The ERT [47] includes an encoding and a recognition phase. During encoding, ten ambiguous social scenarios are displayed, encompassing a title (e.g, “The job interview”), a description (e.g., “You see a job advertised that you’d really like. You apply and are invited to an interview, where you answer the questions as well as you can. Reflecting later, you think that the quality of your answers decided the…”), a word fragment (e.g., “ou_co_e”), and a comprehension question followed by feedback. The word fragment participants have to complete does not resolve the scenario’s ambiguity. During the recognition phase, the scenario title is shown again, along with two target sentences, reflecting negative and positive interpretations (e.g., negative interpretation: “Reflecting later, you realize that your poor answers lost you the job’, positive interpretation: “You think it must have been your clear answers that got you the job”). Interpretation sentences are presented in a random order. Participants indicate how well the target sentence fits the scenario with this title described earlier using a 4-point Likert scale (1 = not at all similar to 4 = very similar). The ERT yields two mean ratings: one for positive sentences and one for negative sentences, thereby reflecting positive and negative interpretations, respectively.

Scenario Rating Task (SRT): To assess neurophysiological correlates of interpretational processes (N400), the SRT was developed. The task includes ambiguous sentence stems presented auditorily. Stems were either social or neutral, followed by congruent (socially positive/neutrally expected) or incongruent (socially negative/neutrally unexpected) endings displayed on the screen. Participants rated how much they expected each ending on a 4-point Likert scale (1 = not at all to 4 = very much). Neutral stems assessed basic N400 (e.g., “Your hand has five …”; congruent: “fingers”, incongruent: “trees”), while social stems targeted SAD-relevant N400 effects (e.g., “When you talk, your cheeks become red. The others find it …”; congruent: “sweet”, incongruent: “embarrassing”). Each trial began with a fixation cross (200 ms), followed by the spoken stem (8000 ms), a jitter (500–750 ms), the outcome word (1000 ms), the rating question, and an intertrial interval (500–1250 ms). The SRT comprised 96 trials (24 per condition: social/neutral × congruent/incongruent) and closely resembled previous ERP-based interpretational bias paradigms [48]. Two versions were used (one for T1, one for T2), counterbalanced across participants.

##### Stress-related psychophysiological assessments

Anagram Task: Stress reactivity to social-evaluative threat was assessed using an anagram task [27]. Framed as a linguistic intelligence test, participants were required to solve 15 anagrams (e.g., “ektrmaesupr” → “supermarket”) within 20 s each while being filmed for evaluation. A beep separated trials, and if unsolved after 10 s, a visible countdown appeared. Regardless of performance, participants received failure feedback after the task. Although solvable, the brief time limit, performance pressure, filming, and feedback were designed to induce social-evaluative stress.

Salivary Cortisol and Alpha-Amylase: Saliva samples to analyze concentrations of cortisol and alpha-amylase (sAA) were collected during both lab assessments. During T1, samples were collected once prior to the administration of the questionnaires. During T2, samples were collected four times: prior to the administration of the questionnaires, pre- and post-anagram, and 25 min post-anagram.

Electrocardiogram (ECG): HR and HRV measures were recorded during both lab assessments. At T1, it was scored for two timepoints: at baseline and after EEG/ECG preparations. At T2, it was scored 6 times: at baseline, after EEG/ECG preparations, pre-, during-, and post-anagram, and recovery (25 min post-anagram). In both sessions, measurement 1 (baseline) was unstandardized in duration, as ECG electrodes were applied first and recording continued until EEG setup was completed. Measurement 2 lasted 8 min (same time as resting-state EEG). At T2, the pre-anagram, post-anagram, and recovery periods each lasted 5 min, during which participants were instructed to remain still. The assessment during the anagram task was unstandardized in duration (up to 5 min). The full procedural sequence is illustrated in Fig. 1A.

Frontal Asymmetry (FAA): Following previous studies [26], resting state EEG was recorded during an 8-minute resting period (in alternating 1-min eyes-open/eyes-closed segments) at both T1 and T2.

##### Other measures

Additional administered measures included: collection of demographic data, expectancy questionnaires, feedback questionnaire, negative effects questionnaire, negative state affect, and accuracy in the anagram task (see Supplementary Materials for details). No measures were collected throughout or between training sessions.

### Data acquisition, processing, and analysis

Full details of data acquisition and pre-processing are provided in the Supplementary Materials.

#### Data analysis

All analyses were performed in R (v4.4.0) and RStudio (v2024.4.1.748) using linear mixed-effects models (LMM) to account for repeated measures. For each outcome, random-intercept and random-slope models were compared using likelihood ratio tests and AIC, with the better-fitting model retained to balance explanatory power and parsimony. Between-group contrasts (change from baseline) were derived from the selected models. Effect sizes (Cohen’s *d*) were calculated from model-based mean differences divided by observed SDs: change-score SDs for between-group and pooled SDs for within-group effects. Hedges’ g correction and 95% CIs were computed using the R package *MBESS* [49].

All tasks and questionnaires had the same 2 × 3 design (group × time), with 2 exceptions: the SRT included 2 additional within-subject variables: valence (neutral, social) and congruence (congruent, incongruent). The ERT included an additional within-subject variable of valence (positive, negative) and an additional assessment, T1.5 (online, between T1 and T2)^1^.

For saliva samples, a specific planned contrast was added to the LMM analysis: pre-anagram vs. post-anagram for sAA and pre-anagram vs. post-anagram+25 mins for cortisol, in line with previous literature on the peak of each measure [50]. For each contrast, the difference between the two groups was also examined.

For ECG data, i.e., mean HR, RMSSD (ln) and high-frequency (HF) power (ln), specific planned contrasts were added to the LMM analysis. These contrasts were in relation to the anagram task in T2 and include: (1) pre-anagram vs. during anagram (measurement 3 vs. 4), and (2) pre-anagram vs. post-anagram (measurement 3 vs. 5; i.e., return to baseline). For each contrast, group differences were also examined. Of note, both HRV analyses included ECG derived respiration (EDR) as a covariate: Because respiration is closely coupled to HRV, particularly HF power, uncontrolled differences in breathing rate across task phases may confound within-subject HRV comparisons. Adjusting for EDR therefore ensured that observed task-related HRV effects could not be attributed to respiration-related variability rather than cardiac autonomic regulation. Results were comparable across analyses, indicating that findings were not driven by statistical control of respiration [51] (see Supplement Materials Tables S2 and S8 for LMM results with and without EDR as a covariate, respectively).

Structural equation modelling (SEM) tested whether post-training IB mediated the effect of group assignment (CBM-I vs. sham) on LSAS at follow-up. Negative IB was modelled as a latent variable combining the negative ERT condition and the SRT social incongruent condition. The outcome variable (LSAS at follow-up) was adjusted for baseline covariates (LSAS, ERT, and SRT at T1). Analyses used the lavaan package [52] with 5000 bootstrap replications for standard errors (SE), and the proportion of the total effect explained by the indirect path was calculated.

Lastly, correlations were run between all T1 variables, with corrections for multiple comparisons using the false discovery rate (FDR) correction (Benjamini–Hochberg procedure). Twenty-one variables were included, resulting in 210 tests and a significance threshold of 0.003795.

## Results

All 88 participants completed all measures at baseline and post-training one week later (i.e., the two lab sessions (T1 and T2). All participants completed all 6 online training sessions, except for one participant who completed 5 sessions (see Supplementary Materials for full details on missing/excluded data, results from additional measures etc.). Participants’ characteristics can be found in Table 1.

**Table 1:** Participants’ characteristics.

Variable	Level	Active (*N* = 44)	Sham (*N* = 44)	*p*-value
Age (Continuous)	*N*	44	44	
	*Mean (SD)*	24.23 (6.24)	25.95 (7.38)	0.239
	Max	56	54	
	Min	19	18	
Age (Binary)	*N*	44	44	
	< 40	43 (97.7%)	42 (95.5%)	0.562
	≥ 40	1 (2.3%)	2 (4.5%)	
T1 LSAS (Continuous)	*N*	44	44	
	*Mean (SD)*	54.32 (22.28)	59.84 (26.56)	0.294
	Max	103	113	
	Min	10	12	
T1 LSAS (Binary)	*N*	44	44	
	< 75	34 (77.3%)	30 (68.2%)	0.344
	≥ 75	10 (22.7%)	14 (31.8%)	
Gender	*N*	44	44	
	Male	7 (15.9%)	7 (15.9%)	1.000
	Female	37 (84.1%)	37 (84.1%)	
Contraception Use (among Female)	*N*	44	44	
	No	27 (61.4%)	24 (54.5%)	0.458
	Yes	10 (22.7%)	13 (29.5%)	
Education	*N*	44	44	
	A-levels	12 (27.3%)	8 (18.2%)	0.315
	Bachelors	1 (2.3%)	3 (6.8%)	0.312
	Higher secondary education (applied)	27 (61.4%)	30 (68.2%)	0.509
	Intermediate secondary education	3 (6.8%)	1 (2.3%)	0.312
	Lower secondary education		2 (4.5%)	0.160
	Masters	1 (2.3%)		0.323
Degree	*N*	44	44	
	Bachelor	21 (47.7%)	26 (59.1%)	0.291
	Master	11 (25%)	5 (11.4%)	0.100
	State exam	7 (15.9%)	3 (6.8%)	0.184
	Other/NA	5 (11.4%)	10 (22.7%)	0.160
Major	*N*	44	44	
	Engineering	2 (4.5%)		0.160
	Humanities	11 (25%)	12 (27.3%)	0.811
	Medicine	5 (11.4%)	1 (2.3%)	0.094
	Natural sciences	3 (6.8%)	3 (6.8%)	1.000
	Psychology	15 (34.1%)	17 (38.6%)	0.662
	Social science	3 (6.8%)	2 (4.5%)	0.650
	NA	5 (11.4%)	9 (20.5%)	0.249
Occupation	*N*	44	44	
	Civil servant	1 (2.3%)		0.323
	Employed	3 (6.8%)	4 (9.1%)	0.698
	Self-employed		1 (2.3%)	0.323
	University student	39 (88.6%)	35 (79.5%)	0.249
	Other	1 (2.3%)	4 (9.1%)	0.172
Nationality	*N*	44	44	
	German	38 (86.4%)	41 (93.2%)	0.297
	Italian	1 (2.3%)		0.323
	Turkish		1 (2.3%)	0.323
	Other	5 (11.4%)	2 (4.5%)	0.243
Relationship Status	*N*	44	44	
	Divorced	1 (2.3%)		0.323
	Married (or comparable)		3 (6.8%)	0.083
	Relationship	25 (56.8%)	23 (52.3%)	0.673
	Single	18 (40.9%)	18 (40.9%)	1.000

*LSAS* liebowitz social anxiety scale.

Analyses revealed that our primary outcome was not significant (i.e., greater reductions in the CBM-I vs. the sham group from T1 to T3). Within-between comparisons for questionnaires, tasks, EEG/ERP, and psychophysiology are summarized in Table 2. Full LMM results can be found in the Supplementary Materials (Table S2).

**Table 2:** Summary of outcomes of all main outcome measures.

Measure	Group		T1 (baseline)	T2 (post-training)	T3 (follow-up)
Liebowitz Social Anxiety Scale (LSAS)	Active	*N*	*n* = 44	*n* = 44	*n* = 42
		*M (SD)*	54.32 (22.28)	48.89 (25.21)	48.26 (28.25)
		Within-group *g* [95% CI]		0.22** [0.05, 0.41]	0.21* [0.00, 0.42]
	Sham	*N*	*n* = 44	*n* = 44	*n* = 39
		*M (SD)*	59.84 (26.56)	55.98 (28.99)	51.49 (29.30)
		Within-group *g* [95% CI]		0.14 [−0.03, 0.30]	0.29** [0.09, 0.49]
	Between-group g [95% CI]		−0.22 [−3.02, 0.91]	−0.11 [−0.52, 0.31]	0.08 [−0.36, 0.52]
Social Phobia and Anxiety Inventory (German; SPAI-G)	Active	*N*	*n* = 44	*n* = 44	*n* = 41
		*M (SD)*	68.33 (20.22)	64.66 (21.56)	61.83 (26.39)
		Within-group *g* [95% CI]		0.17* [0.04, 0.31]	0.28** [0.09, 0.48]
	Sham	*N*	*n* = 44	*n* = 44	*n* = 41
		*M (SD)*	67.42 (19.75)	66.28 (21.99)	62.99 (26.22)
		Within-group *g* [95% CI]		0.05 [−0.07, 0.18]	0.16 [−0.04, 0.36]
	Between-group *g* [95% CI]		0.05 [−1.75, 2.17]	−0.28 [−0.70, 0.14]	−0.25 [−0.68, 0.19]
Brief Fear of Negative Evaluation (BFNE)	Active	*N*	*n* = 43	*n* = 44	*n* = 44
		*M (SD)*	43.77 (9.50)	41.84 (10.04)	40.18 (11.80)
		Within-group *g* [95% CI]		0.18* [0.05, 0.32]	0.32** [0.09, 0.55]
	Sham	*N*	*n* = 44	*n* = 43	*n* = 41
		*M (SD)*	42.27 (9.56)	42.09 (9.81)	42.15 (9.74)
		Within-group *g* [95% CI]		0.00 [−0.14, 0.14]	−0.04 [−0.23, 0.14]
	Between-group *g* [95% CI]		0.16 [−1.23, 2.69]	−0.42 [−0.85, 0.01]	−0.57** [−1.01, −0.13]
Depression, Stress and Anxiety (DASS-21)	Active	*N*	*n* = 44	*n* = 43	*n* = 42
		*M (SD)*	18.07 (11.12)	17.02 (9.83)	16.05 (13.49)
		Within-group *g* [95% CI]		0.12 [−0.10, 0.35]	0.17 [−0.07, 0.41]
	Sham	*N*	*n* = 44	*n* = 44	*n* = 40
		*M (SD)*	17.55 (11.75)	14.93 (9.58)	15.25 (10.98)
		Within-group *g* [95% CI]		0.24* [0.00, 0.48]	0.10 [−0.19, 0.39]
	Between-group *g* [95% CI]		0.05 [−1.75, 2.17]	0.16 [−0.26, 0.58]	−0.04 [−0.48, 0.39]
Encoding Recognition Task (ERT – Negative Condition)	Active	*N*	*n* = 44	*n* = 44	*n* = 44
		*M (SD)*	2.31 (0.56)	1.85 (0.48)	1.82 (0.57)
		Within-group *g* [95% CI]	NA	−0.79*** [−7.40, −2.97]	−0.88*** [−8.13, −3.55]
	Sham	*N*	*n* = 44	*n* = 44	*n* = 41
		*M (SD)*	2.16 (0.56)	2.17 (0.54)	2.15 (0.59)
		Within-group *g* [95% CI]	NA	0.04 [−1.69, 2.16]	−0.05 [−2.27, 1.58]
	Between-group g [95% CI]		0.27 [−0.15, 0.68]	−0.62** [−1.04, −0.19]	−0.56* [−0.99, −0.13]
Encoding Recognition Task (ERT – Positive Condition)	Active	*N*	*n* = 44	*n* = 44	*n* = 44
		*M (SD)*	2.12 (0.58)	2.68 (0.54)	2.74 (0.63)
		Within-group g [95% CI]	NA	0.92*** [3.79, 8.43]	0.93*** [3.82, 8.47]
	Sham	*N*	*n* = 44	*n* = 44	*n* = 41
		*M (SD)*	2.03 (0.58)	2.37 (0.55)	2.22 (0.66)
		Within-group g [95% CI]	NA	0.71*** [2.50, 6.83]	0.30 [−0.07, 3.87]
	Between-group g [95% CI]		0.16 [−0.26, 0.57]	0.58** [0.15, 1.00]	0.80*** [0.35, 1.23]
Scenario Rating Task (SRT - Ratings) - Neutral-Congruent	Active	*N*	*n* = 44	*n* = 44	
		*M (SD)*	3.94 (0.09)	3.96 (0.06)	
		Within-group g [95% CI]		−0.22 [−0.63, 0.19]	
	Sham	*N*	*n* = 44	*n* = 44	
		*M (SD)*	3.90 (0.12)	3.95 (0.12)	
		Within-group *g* [95% CI]		−0.37 [−0.80, 0.04]	
	Between-group *g* [95% CI]		0.37 [−0.25, 3.71]	−0.20 [−0.62, 0.22]	
Scenario Rating Task (SRT - Ratings) - Neutral-Incongruent	Active	*N*	*n* = 44	*n* = 44	
		*M (SD)*	1.03 (0.07)	1.01 (0.05)	
		Within-group *g* [95% CI]		0.27 [−0.05, 0.61]	
	Sham	*N*	*n* = 44	*n* = 44	
		*M (SD)*	1.03 (0.06)	1.03 (0.09)	
		Within-group *g* [95% CI]		0.01 [−0.27, 0.29]	
	Between-group g [95% CI]		0.03 [−1.83, 2.09]	−0.40 [−0.82, 0.02]	
Scenario Rating Task (SRT - Ratings) - Social-Congruent (Positive)	Active	*N*	*n* = 44	*n* = 44	
		*M (SD)*	2.45 (0.37)	2.78 (0.36)	
		Within-group *g* [95% CI]		−0.88*** [−1.21, −0.57]	
	Sham	*N*	*n* = 44	*n* = 44	
		*M (SD)*	2.38 (0.49)	2.61 (0.43)	
		Within-group *g* [95% CI]		−0.49*** [−0.78, −0.22]	
	Between-group g [95% CI]		0.15 [−1.24, 2.69]	−0.08 [−0.50, 0.34]	
Scenario Rating Task (SRT - Ratings) - Social-Incongruent (Negative)	Active	*N*	*n* = 44	*n* = 44	
		*M (SD)*	2.27 (0.49)	1.96 (0.47)	
		Within-group *g* [95% CI]		0.64*** [0.39, 0.91]	
	Sham	*N*	*n* = 44	*n* = 44	
		*M (SD)*	2.28 (0.51)	2.21 (0.55)	
		Within-group *g* [95% CI]		0.12 [−0.06, 0.29]	
	Between-group *g* [95% CI]		−0.02 [−2.03, 1.89]	−0.08 [−0.50, 0.33]	
Scenario Rating Task (SRT - N400) - Neutral-Congruent	Active	*N*	*n* = 34	*n* = 31	
		*M (SD)*	1.03 (1.59)	0.99 (1.25)	
		Within-group *g* [95% CI]		0.04 [−0.46, 0.54]	
	Sham	*N*	*n* = 29	*n* = 28	
		*M (SD)*	0.37 (1.54)	0.42 (1.51)	
		Within-group *g* [95% CI]		−0.10 [−0.78, 0.57]	
	Between-group *g* [95% CI]		0.42 [−0.31, 3.65]	−0.04 [−0.62, 0.54]	
Scenario Rating Task (SRT - N400) - Neutral-Incongruent	Active	*N*	*n* = 34	*n* = 31	
		*M (SD)*	0.67 (1.04)	0.31 (1.01)	
		Within-group *g* [95% CI]		0.46 [0.05, 0.89]	
	Sham	*N*	*n* = 29	*n* = 28	
		*M (SD)*	−0.25 (1.01)	−0.09 (1.15)	
		Within-group *g* [95% CI]		−0.25 [−0.83, 0.31]	
	Between-group *g* [95% CI]		0.89 [1.49, 5.61]	−0.08 [−0.66, 0.50]	
Scenario Rating Task (SRT - N400) - Social-Congruent (Positive)	Active	*N*	*n* = 34	*n* = 31	
		*M (SD)*	0.92 (1.35)	0.72 (1.16)	
		Within-group *g* [95% CI]		0.17 [−0.37, 0.71]	
	Sham	*N*	*n* = 29	*n* = 28	
		*M (SD)*	0.00 (1.54)	0.26 (1.78)	
		Within-group *g* [95% CI]		−0.17 [−0.82, 0.46]	
	Between-group *g* [95% CI]		0.63 [0.50, 4.52]	−0.04 [−0.62, 0.53]	
Scenario Rating Task (SRT - N400) – Social -Incongruent (Negative)	Active	*N*	*n* = 34	*n* = 31	
		*M (SD)*	0.81 (1.37)	0.38 (1.04)	
		Within-group *g* [95% CI]		0.35 [−0.18, 0.90]	
	Sham	*N*	*n* = 29	*n* = 28	
		*M (SD)*	0.10 (1.57)	0.25 (1.24)	
		Within-group *g* [95% CI]		−0.15 [−0.74, 0.43]	
	Between-group *g* [95% CI]		0.48 [−0.07, 3.91]	−0.05 [−0.63, 0.53]	
Frontal Alpha Asymmetry	Active	*N*	*n* = 42	*n* = 44	
		*M (SD)*	0.04 (0.16)	0.04 (0.19)	
		Within-group *g* [95% CI]		−0.08 [−0.40, 0.25]	
	Sham	*N*	*n* = 42	*n* = 43	
		*M (SD)*	0.03 (0.23)	0.06 (0.20)	
		Within-group *g* [95% CI]		−0.08 [−0.51, 0.35]	
	Between-group *g* [95% CI]		0.03 [−1.83, 2.09]	−0.10 [−0.53, 0.33]	
Saliva - Cortisol	Active	*N*	*n* = 41	*n* = 170	
		*M (SD)*	3.78 (2.30)	2.79 (1.75)	
		Within-group g [95% CI]		0.50** [0.08, 0.94]	
	Sham	*N*	*n* = 33	*n* = 164	
		*M (SD)*	4.28 (2.54)	3.32 (2.02)	
		Within-group *g* [95% CI]		0.44* [−0.03, 0.92]	
	Between-group *g* [95% CI]		−0.21 [−2.85, 1.08]	0.00 [−0.46, 0.46]	
Saliva - Alpha-Amylase (sAA)	Active	*N*	*n* = 42	*n* = 170	
		*M (SD)*	146.07 (98.48)	160.01 (85.48)	
		Within-group *g* [95% CI]		−0.11 [−0.55, 0.33]	
	Sham	*N*	*n* = 40	*n* = 164	
		*M (SD)*	119.05 (91.60)	143.39 (91.83)	
		Within-group *g* [95% CI]		−0.56 [−1.00, −0.14]	
	Between-group *g* [95% CI]		0.28 [−0.69, 3.25]	−0.05 [−0.48, 0.38]	
Electrocardiogram (ECG) - mean HR	Active	*N*	*n* = 85	*n* = 254	
		*M (SD)*	75.08 (10.60)	73.32 (11.70)	
		Within-group *g* [95% CI]		0.37* [−0.03, 0.78]	
	Sham	*N*	*n* = 82	*n* = 244	
		*M (SD)*	75.38 (13.23)	71.98 (11.85)	
		Within-group *g* [95% CI]		0.50*** [0.07, 0.95]	
	Between-group *g* [95% CI]		−0.03 [−2.12, 1.80]	0.09 [−0.33, 0.51]	
Electrocardiogram (ECG) - RMSSD (log)	Active	*N*	*n* = 85	*n* = 233	
		*M (SD)*	3.76 (0.50)	3.72 (0.50)	
		Within-group *g* [95% CI]		−0.05 [−0.43, 0.34]	
	Sham	*N*	*n* = 82	*n* = 234	
		*M (SD)*	3.63 (0.46)	3.70 (0.44)	
		Within-group *g* [95% CI]		−0.46* [−0.91, −0.03]	
	Between-group *g* [95% CI]		0.24 [−0.40, 3.53]	−0.13 [−0.56, 0.30]	
Electrocardiogram (ECG) - HF-Power (log)	Active	*N*	*n* = 79	*n* = 226	
		*M (SD)*	6.51 (0.91)	6.50 (0.98)	
		Within-group *g* [95% CI]		−0.03 [−0.43, 0.36]	
	Sham	*N*	*n* = 79	*n* = 225	
		*M (SD)*	6.36 (0.78)	6.44 (0.82)	
		Within-group *g* [95% CI]		−0.44 [−0.92, 0.02]	
	Between-group *g* [95% CI]		0.16 [−0.97, 2.96]	−0.07 [−0.50, 0.37]	

# marks the primary outcome (LSAS at T1–T3 between groups). T1 is the reference point for all comparisons: between-group comparisons at T1 reflect baseline group differences, while later comparisons reflect differences relative to T1. Complete ERT results (including T1.5) are available in the Supplementary Materials. The interval between measurement points was 1 week. Positive within-group effect sizes indicate improvement; negative between-group effect sizes indicate superiority of CBM-I over sham, except for ERT (Positive) and SRT (Ratings) – Social-Congruent (Positive), where the interpretation is reversed. *p* < 0.05, *p* < 0.01, *p* < 0.001.

*M* mean, *SD* standard deviation.

### Encoding Recognition Task (ERT)

As shown in Table 2, from T1 to T2 and to T3, positive interpretations increased and negative interpretations decreased only in the CBM-I group, while the sham group showed increased positive interpretation only from T1 to T2. Group differences were significant for both valences. All time × valence and time × group × valence interactions were significant (*p* < 0.001; see the Supplementary Materials for the inclusion of T1.5).

### Scenario Rating Task (SRT) – subjective ratings

As shown in Table 2, from T1 to T2, positive interpretations increased in both groups, while negative interpretations decreased only in the CBM-I group. However, between-group differences were non-significant. Neutral interpretations did not change across time or groups, serving as a manipulation check. The SRT has two additional variables: valence and congruence. While no main or interaction effects of group emerged (all *p*s > 0.07), of the two-way interactions, only time × valence interaction was significant (*p* = 0.002), indicating that the increase in expectedness from T1 to T2 was larger for social than neutral stimuli. The congruence × valence interaction was also significant (*p* < 0.001), reflecting a smaller congruence effect for social trials. Finally, the three-way interaction between time, congruence and valence was significant (*p* < 0.001), indicating that the congruence-by-valence pattern differed between T1 and T2. No other interactions reached significance (all *p*s > 0.072; see Figure S3 in Supplementary Materials).

### Scenario Rating Task (SRT) - N400

While the N400 does not show any effects of time, group, or their interaction (all *p*s > 0.056), it does mirror the same findings as the subjective ratings, in the same rank order: neutral-incongruent sentences (i.e., most unexpected condition) showed the most negative amplitudes, followed by social-negative sentences, followed by social-positive sentences, and the most positive amplitudes (i.e., most expected condition) were exhibited for the neutral-congruent sentences. All higher-order interactions, including the four-way interaction, were non-significant (all *p* > 0.24; see Supplementary Materials for an exploratory simplified N400 analysis, as well as an exploratory analysis of the time window between 200–300).

### Salivary measures

Contrary to hypotheses, results show a significant decrease in cortisol post- compared to pre-anagram. sAA levels did not change (see Table 3).

**Table 3:** Planned contrast results for salivary measures.

Measure	Group		Pre-Anagram vs. Post-Anagram	Pre-anagram vs Post-anagram + 25 mins
Alpha-Amylase	Active	*N*	*n* = 43	
		*M (SD)*	161.60 (87.21), 168.05 (92.45)	
		Within-group *g* [95% CI]	0.07 [−1.30, 2.56]	
	Sham		*n* = 40	
		*M* (*SD*)	138.64 (93.56), 151.72 (93.52)	
		Within-group g [95% CI]	0.14 [−0.68, 3.20]	
		Between-group *g* [95% CI]	−0.10 [−0.53, 0.33]	
Cortisol	Active	*N*		*n* = 43
		*M (SD)*		2.56 (1.49), 2.17 (1.52)
		Within-group *g* [95% CI]		−0.26** [−5.05, −0.99]
	Sham			*n* = 41
		*M (SD)*		3.26 (1.92), 2.65 (1.83)
		Within-group *g* [95% CI]		−0.32** [−5.29, −1.19]
		Between-group *g* [95% CI]		0.21 [−0.22, 0.63]

* *p* < 0.05; ** *p* < 0.01; *** *p* < 0.001.

### Electrocardiogram (ECG)

Overall, ECG results indicated increased sympathetic activity (reflected in higher mean HR) and decreased parasympathetic activity (reflected in lower HRV indices) during the anagram task compared to pre-task. Furthermore, HR and HRV values returned to baseline, as post-anagram levels did not significantly differ from pre-task. However, no group differences emerged for any contrast (see Table 4 for full details).

**Table 4:** Planned contrast results for ECG measures.

	Group		Pre-Anagram vs. During-Anagram	Pre-Anagram vs. Post-Anagram
Mean HR	Active		*n* = 43	
		*M (SD)*	70.51 (11.42), 73.47 (11.60)	
		Within-group *g* [95% CI]	0.25*** [1.40, 5.52]	
	Sham		*n* = 40	
		*M (SD)*	69.29 (11.21), 74.03 (12.17)	
		Within-group *g* [95% CI]	0.40*** [3.55, 8.19]	
	Between-group *g* [95% CI]		−0.34 [−0.76, 0.10]	
	Active			*n* = 43
		*M (SD)*		70.51 (11.42), 71.27 (11.55)
		Within-group *g* [95% CI]		0.06 [−0.56, 3.34]
	Sham			*n* = 41
		*M (SD)*		69.74 (11.21), 69.98 (11.54)
		Within-group *g* [95% CI]		0.02 [−1.46, 2.40]
	Between-group *g* [95% CI]			0.15 [−0.28, 0.57]
RMSSD (log)	Active		*n* = 38	
		*M (SD)*	3.78 (0.54), 3.65 (0.56)	
		Within-group *g* [95% CI]	−0.22* [−4.60, −0.58]	
	Sham		*n* = 37	
		*M (SD)*	3.74 (0.51), 3.56 (0.53)	
		Within-group *g* [95% CI]	−0.34*** [−6.48, −2.15]	
	Between-group *g* [95% CI]		0.20 [−0.25, 0.65]	
	Active			*n* = 38
		*M (SD)*		3.78 (0.54), 3.73 (0.53)
		Within-group *g* [95% CI]		−0.08 [−3.14, 0.74]
	Sham			*n* = 38
		*M (SD)*		3.73 (0.51), 3.72 (0.50)
		Within-group *g* [95% CI]		−0.02 [−2.21, 1.63]
	Between-group *g* [95% CI]			−0.17 [−0.62, 0.28]
HF-Power (log)	Active		*n* = 37	
		*M (SD)*	6.63 (1.10), 6.38 (1.11)	
		Within-group *g* [95% CI]	−0.22* [−4.32, −0.34]	
	Sham		*n* = 35	
		*M (SD)*	6.50 (1.02), 6.14 (1.05)	
		Within-group *g* [95% CI]	−0.35*** [−6.03, −1.77]	
	Between-group *g* [95% CI]		0.19 [−0.26, 0.65]	
	Active			*n* = 37
		*M (SD)*		6.63 (1.10), 6.49 (1.10)
		Within-group *g* [95% CI]		−0.13* [−4.01, −0.05]
	Sham			*n* = 35
		*M (SD)*		6.44 (1.02), 6.40 (0.86)
		Within-group *g* [95% CI]		−0.04 [−2.53, 1.32]
	Between-group *g* [95% CI]			−0.26 [−0.72, 0.20]

* *p* < 0.05; ** *p* < 0.01; *** *p* < 0.001.

### Mediation

Both ERT and SRT showed strong standardized loadings on the latent IB factor (0.80 and 0.94, respectively), indicating that the factor explained about 64% of the variance in ERT and 89% of the variance in SRT, with 36% and 11% remaining as indicator‑specific variance, respectively. The tasks were strongly correlated (r = 0.76, 95% CI [0.64, 0.84]), supporting their convergent validity.

The mediation model revealed that group assignment significantly predicted negative IB at T2, with participants in the sham condition showing greater negative IB than those in the CBM-I condition (β = 0.22, *SE* = 0.10, *p* = 0.032). In turn, higher levels of IB significantly predicted higher LSAS scores at T3 (β = 28.90, *SE* = 5.03, *p* < 0.001). The direct path from group to LSAS scores was also significant (β = −7.80, *SE* = 3.34, *p* = 0.019), as well as the indirect effect of group on LSAS through IB (β = 6.40, *SE* = 2.74, *p* = 0.020, 95% CI [1.17, 11.95]). This pattern indicates partial mediation, such that the effect of group on social anxiety outcomes at follow-up operated partially through its impact on IB.

Of note, the current model showed poor fit indices (*χ²*(7) = 102.58, *p* < 0.001, CFI = 0.65, TLI = 0.26, RMSEA = 0.41, 90% CI [0.34, 0.48], SRMR = 0.23). Thus, an additional exploratory model was estimated without T1 controls, which demonstrated substantially better fit (*χ²*(1) = 5.98, *p* = 0.014, CFI = 0.95, TLI = 0.71, RMSEA = 0.25, 90% CI [0.09, 0.45], SRMR = 0.04). Results for this model are provided in the Supplementary Materials.

### T1 intercorrelations

**Fig. 2:**
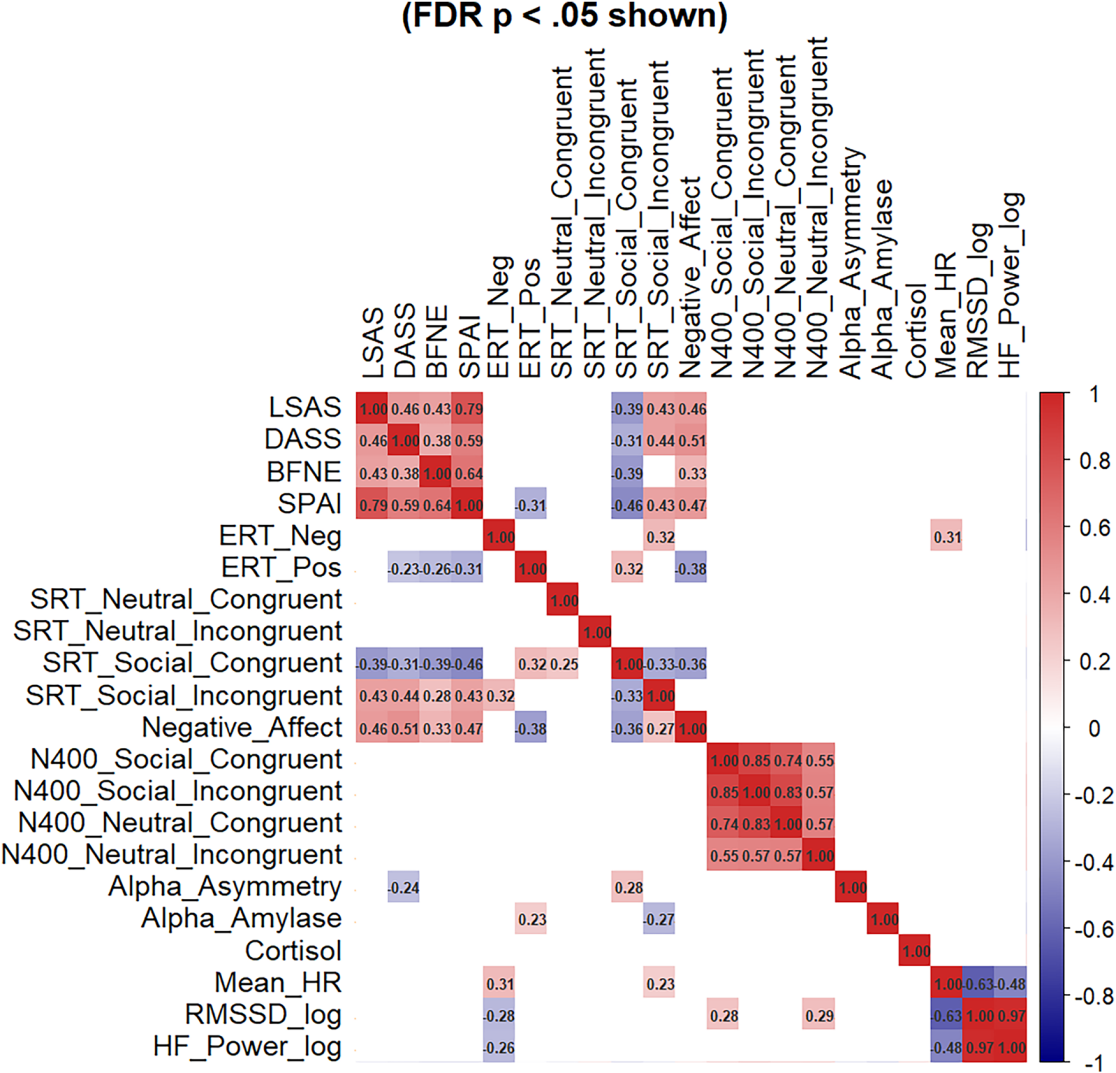
Heatmap visualization of intercorrelations at T1 (FDR corrected). LSAS liebowitz social anxiety scale, SPAI social phobia and anxiety inventory, BFNE brief fear of negative evaluation, DASS depression, anxiety and stress scale, ERT encoding recognition task, ERT_neg negative ERT condition, ERT_pos positive ERT condition, HR heart rate.

A correlations matrix revealed significant correlations between tasks and questionnaires at T1 (see Fig. 2).

### Additional results

Results of reliability analysis and all other measures can be found in the Supplementary Materials.

## Discussion

This preregistered RCT is the first to provide a comprehensive assessment of the effects of multisession CBM-I on psychopathology symptoms, cognitive, neural, and psychophysiological effects. Specifically, participants with high levels of social anxiety took part in a multisession CBM-I procedure, with the active group training positive interpretations.

Considering changes in LSAS scores from pre-training to follow-up as the primary outcome, we expected greater reductions in the CBM-I versus the sham training group. While LSAS scores reduced across sessions, training did not affect this change. While these results are not in line with our expectations, the mediation analysis does show that negative IB post-training mediated the effect of training on LSAS at follow-up. Specifically, at post-training, the CBM-I group showed less negative IB, which in turn, led to lower follow-up LSAS scores. This finding supports the proposed mechanism of change in CBM-I by demonstrating that reductions in negative IB can account for subsequent decreases in social anxiety symptoms.

Secondary outcomes included other social anxiety-relevant questionnaires, i.e., the BFNE, DASS-21, SPAI-G, and the ERT and SRT to reflect IB. Additional measures comprised neural and psychophysiological measures including salivary sAA and cortisol, HR and HRV measures, N400 and FAA. However, results on these secondary outcomes were inconsistent. IB changed from pre- to post-training: interpretations in the ERT became more positive and less negative immediately after training, and more so in the CBM-I group. While SRT showed no significant group differences, only the CBM-I group showed less negative IB from T1 to T2. The SRT’s results were consistent in subjective ratings and N400 amplitudes. Specifically, both outcomes clearly distinguished congruent from incongruent neutral sentences, while responses to positive and negative emotional sentences fell between the most and least expected. Thus, neural processing of emotional sentences differed from neutral ones, as what is objectively (un)expected is not the same as what is emotionally (un)expected. As for social anxiety symptoms, while LSAS and SPAI-G decreased in both groups, BFNE was reduced at follow-up only in the CBM-I group.

During and following stress induction, across both groups, self-reported negative affect and sympathetic activity (HR) increased while parasympathetic activity (HRV) decreased. Thus, while the task was stress-inducing, it did not appear sensitive enough to reveal group differences. This may indicate that the induced stress was unrelated to the trained cognition, or that CBM-I was insufficient to produce differential stress responses. Cortisol and sAA, however, were not as sensitive to the stress task: Contrary to hypotheses, cortisol levels decreased after the task and sAA levels remained unchanged. Thus, the stressor may have been too weak to activate the HPA axis. As cortisol levels decrease over the day, the observed pattern may be expected, in addition to the reduction of pre-anagram anticipatory stress [53]. Future studies could employ more socially relevant stress-inducing tasks that better match the cognitions targeted during CBM-I, such as the Trier Social Stress Test (TSST), which reliably activates the HPA axis and produces robust cortisol increases [54].

Although the LSAS was the primary outcome, only BFNE scores changed after CBM-I, indicating that these questionnaires capture different aspects of social anxiety: the LSAS targets specific performance and interaction fears, whereas the BFNE measures broader concerns about negative evaluation. CBM-I may have targeted only the latter profile, thus emphasizing the need to clarify measure–training alignment. Future studies could also explore the bivalent model distinguishing fear of negative from fear of positive evaluation, as both contribute to social anxiety [55].

Our results support several studies examining the multimodal effects of training using ERPs. For instance, a previous study examined the effects of a single-session imagery-enhanced interpretation training in worry [56]. While both online (i.e., reaction time based) and offline interpretations (i.e., ratings) were found to be more positive following training compared to the sham condition, no differences emerged on neurophysiological markers during the online task. Thus, neural measures may be insensitive to CBM-I or may need more sessions to show effects.

Although no ERP changes were found, ERPs distinguished neural processing of emotional versus neutral sentences. The few studies examining IB using ERPs did not include neutral sentences but socially-relevant sentences with benign outcomes [19]. The clearly distinct expected vs. unexpected neutral sentences indicate that the N400 indeed reflects expectancy violation in our newly developed SRT. However, the distinction between positive vs. negative social sentences was not as clear. Specifically, social anxiety is characterized by multiple biased cognitions, and different sentences may elicit different responses. In this RCT, stimuli spanned various SAD-related cognitions, and so future studies could consider more tailored cognitions when assessing (subtle) ERP effects in the context of positive and negative social sentences. Future studies could also examine trial-level EEG, instead of the averaged ERP, to examine whether within-subject variability is associated with a specific individual versus combined cognition.

The ERP findings should be interpreted in the study’s specific context. First, the final ERP sample was smaller than planned due to participant exclusion during preprocessing, which limited statistical power. Thus, replication with larger samples is warranted. Second, although a positive-going divergence emerged around 200–300 ms, this timing aligns with early attentional components (e.g., P2/early P3) and is functionally distinct from the centro-parietal N400 effect observed between 300 and 450 ms. While this RCT focused on the linguistically driven N400 as a marker of IB, future studies may also examine earlier, more bottom-up components.

Prior studies similarly identified IB as a mediator, such that the effect of CBM-I on LSAS was partially mediated via IB [57]. Similar findings were obtained when CBM-I targeted reductions in repetitive negative thinking in generalized anxiety disorder and depression [58] and in PTSD [16]. Together, these results suggest that IB plays a role as a partial mediator in reducing psychopathology symptoms. However, two mediation models were estimated: one with baseline LSAS, ERT and SRT as covariates, and a more parsimonious one without covariates. While the latter showed a better fit, both models showed a significant mediation effect. Importantly, all mediation results should be interpreted with caution, due to a relatively small sample size.

The reported results should be interpreted in light of several limitations. First, while the SRT showed promising findings, it is still a newly developed task that needs further validation. Second, LSAS and SPAI-G decreased from T1 to T2 across both groups, which may suggest either a placebo effect or a true effect of the sham training. However, this finding should be qualified by the ERT and SRT becoming more positive (and the SRT also less negative) only in the CBM-I group. Of note, the current RCT was not an efficacy trial but rather a mechanistic study examining mechanisms in multimodal outcomes. Hence, CBM-I may still have been too brief to sufficiently shift bias and impact symptoms. Further, sample characteristics need to be taken into account. While it was planned to recruit participants with high levels of social anxiety, LSAS baseline were lower than those in previous CBM studies [59, 60]. Thus, the RCT effects may have been attenuated by participants’ relatively low social anxiety.

Overall, results underscore the importance of multimodal assessments when evaluating CBM-I effects and mechanisms. Although measures varied in sensitivity, T1 correlations showed strong convergent validity within questionnaires and between tasks and questionnaires. Of note, all questionnaires correlated positively with agreement to negative SRT sentences and negatively with positive ones, further supporting the SRT’s validity as an IB measure. For the ERT, only the positive condition correlated negatively with questionnaires, suggesting that social anxiety may reflect reduced positive interpretations rather than strong negative ones. Negative ERT further correlated positively with HR and negatively with HRV, linking negative IB with autonomic activation and highlighting psychophysiological activity as potential IB marker [19]. Together, both tasks emphasize that positive and negative IBs may represent qualitatively distinct, not opposing, processes [61].

To conclude, the present comprehensive RCT provides an in-depth examination of multimodal effects of CBM-I on social anxiety. While results were not always consistent, they do emphasize that social anxiety may have different expressions requiring different measurements. Importantly, reduction in social anxiety was mediated by reduced negative IB levels following CBM-I, further emphasizing the role of IBs in psychopathology in general and in social anxiety in particular.

## Supplementary information

Supplementary Materials

## Data Availability

Anonymized data, along with pre-processing and analysis pipelines, are available on the Open Science Framework (OSF): https://osf.io/e254p/?view_only=13bc5c6b202b4083a86917100a351fa6.

## Footnotes

1 Although the pre-registered protocol proposed computing bias indices as difference scores (e.g., incongruent – congruent for the SRT; negative – positive for the ERT), we retained the raw values instead. This allowed us to capture both positive and negative biases, examine the four distinct N400 waveforms in the SRT (social/neutral × congruent/incongruent), and test for differential valence effects in both tasks without losing information through subtraction.

## Code availability

This randomized controlled trial (RCT) was preregistered on ClinicalTrials.gov (Identifier: NCT05798078; https://clinicaltrials.gov/study/NCT05798078), and all methods and planned analyses were preregistered on the Open Science Framework (OSF). Scripts used for data analysis can also be found on the OSF (same link as “Data Availability”). Unless stated otherwise, all methodological details are in line with the preregistration.
