# Supplementary material for: Multi-session CBM-I for social anxiety: examining psychopathology, cognitive, neural, and psychophysiological effects in a randomized controlled trial
Source: Transl Psychiatry. 2026 May 22;16:279. doi: 10.1038/s41398-026-04122-2 (PMC13197415; doi:10.1038/s41398-026-04122-2)
Supplement: Supplementary file 1 — Supplementary Materials [file 41398_2026_4122_MOESM1_ESM.docx]

**Supplementary Materials**

# **Methods**

## **Participants**

### **Eligibility Criteria**

Study eligibility were determined based on the following inclusion and exclusion criteria:

General inclusion criteria:

1. Provides informed consent
2. Sufficient German language skills to complete the experimental tasks and questionnaires.
3. Aged between 18 and 65 years.
4. Scoring ≥ 52 on the SPAI-G^1,2^, indicating elevated levels of social anxiety.
5. Lives within reasonable travelling distance of the research centre.

Exclusion criteria:

1. Current psychotherapeutic treatment or psychotherapeutic treatment completed within the last 6 months prior to study enrolment.
2. Current substance misuse or dependency (apart from Nicotine).
3. Acute suicidality or self-harm.
4. Symptoms of a psychotic or bipolar disorder.
5. Presence of a somatic condition that could systematically affect cortisol levels (in particular: Pregnancy and lactation, adrenal dysfunction, thyroid dysfunction, pituitary dysfunction).
6. Presence of a somatic condition that could systematically affect brain physiology (current or anamnestic neurological disorders, in particular: anamnestic traumatic brain injury, epilepsy, multiple sclerosis, brain tumors).
7. Presence of a somatic condition that could systematically affect peripheral physiological measures, in particular cardiovascular diseases (e.g., cardiac arrhythmias, circulatory diseases such as hypertension).
8. Sensitivity or alteration of skin surface providing contraindication for EEG or periphysiological measures (in particular: baldness, dreadlocks, open wounds on the head or facial surface, skin conditions that cause particular sensitivity to gels and creams).
9. Intake of psychotropic medication that cannot be interrupted during study duration or change in psychotropic medication within the 8 weeks before starting the study (except: antidepressants in unaltered dosage).
10. Left handedness.

Please note, although current or recent treatment was an exclusion criterion for taking part in the study, whether participants started or received treatment during their participation was not assessed.

### ***Details of Randomization***

Randomization was stratified by gender in combination with use of the contraceptive pill (female, non-contraceptive pill vs. female or non-binary / other, taking contraceptive pill vs. male / non-binary / other), baseline score on the LSAS-SR (< 75 vs. ≥ 75), and age (40 vs. ≥ 40) to match trial arms on these variables. Due to the large number of strata relative to the sample size, fixed block lengths of 2 were used. The random sequences were generated using a true random number generator (www.random.org) by a researcher not involved in any participant contact or the running of the trial (SEB). Randomization was implemented via a web-based interface using Qualtrics software (programmed by SEB). When a participant was due to be randomized, the assessing researcher entered the participant ID, age, gender, and LSAS score into the Qualtrics interface. After submitting this information, the participant’s allocation was displayed on the computer screen, at which point the participant was considered randomized into the trial. For the purpose of allocation concealment, allocation occurred only after participants had completed baseline measures during the pre-assessment, and researchers involved in day-to**-**day running of the trial and with participant contact had no access to the randomization sequence (which was stored within the Qualtrics system and was only accessible to SEB) and were not informed about details of its construction such as block length. Details of the randomization, including date and time, were saved by Qualtrics, thus providing a record of the allocation process.

### ***Blinding***

In line with its focus on mechanisms, the study was not presented to participants as evaluating interventions but rather examining the relationship between thinking styles and anxiety. Information about the online training was presented in a general form that related to both versions, e.g., reading sentences and completing word fragments, and hence participants were blind to their allocation. Researchers conducting the testing sessions did not receive information about whether the participants were in the active or control group. At randomization, the researcher was presented with the information that the participant should complete “version 1” or “version 2” of the program, and used this to set the participant up for the correct training version. As they did not observe the training, they were unaware of which version was the active and which the sham training. However, success of blinding was not formally assessed. We note that the trial registration states that the trial is single blind (participants), as at the point of registration the feasibility of blinding researchers in this way was unknown.

## **Measures**

### ***Negative State Affect***

Negative state affect was measured at relevant time points throughout the assessments. Specifically, during the first lab session, 3 assessments took place: (1) at baseline, (2) after EEG/ECG preparations, (3) after answering questionnaires. During the second lab session, 6 assessments took place: same three timepoints + (4) pre-stressor (anagram task), (5) post-stressor, and (6) during recovery phase (i.e., approx. 25 minutes post-stressor). State affect was measured using an adapted 7-item scale, ranging from 1-5, developed by Becker et al. (2016). This scale includes the following items: “I feel… tense / sad / anxious / confident / relaxed / happy / relieved”, with each item being judged via a 5-point Likert scale (1 not at all – 5 very).

### ***Expectancy Questionnaire (EQ)***

Expectancy effects were assessed once, prior to training, using the three expectancy items from the Credibility and Expectancy Questionnaire^3^. The three questions ask about expected improvement in anxiety due to participation in the study’s training. Questions 1 and 3 are on a scale of 0-100 while question 2 is on a scale of 1-9.

### ***Negative Effects Questionnaire (NEQ)***

To assess potential negative effects and adverse events linked to the study, the short (20-item) version of the Negative Effects Questionnaire was used^4^. The participants are asked to read each incident and indicate if it had occurred to them (yes/no). If ‘yes’, they are asked to rate the negative extent to which the incident has affected them, on a scale of 0-4. Also, if ‘yes’, they are asked to indicate the reason which they believe caused the incident (participation in the study/other circumstances). The questionnaire was adapted for the purpose of the study with terms like ‘therapy’ and ‘therapist’ changed to e.g., ‘study’ and ‘researchers’. The NEQ was applied once, at follow-up.

### ***Feedback Questionnaire***

To assess any feedback concerning the CBM-I program, a CBM-I specific feedback questionnaire was employed, as used by a previous study^5^. First, participants rated the training’s impact on their symptoms on a scale from -10 to +10 where lower values indicate a more positive effect. Using a 9-point Likert scale, participants are then asked to rate how easy they found the training, their satisfaction with the training and its properties (e.g., length and speed), how exhausting the training was, the potential to recommend it to a friend, the potential to use it again in the future, and positive impacts on mood, thoughts, and behaviour. Finally, participants are asked whether they experienced any adverse events and to describe them, what they think the goal of the training was, and if it had a positive effect on their symptoms, what they expect to be the reason for that. The feedback questionnaire was applied once, at follow-up.

## **Data Acquisition**

All in-lab data were collected using Inquisit (Millisecond Software, v4.0.10, 2016), except for the SRT/EEG measurements, where behavioral data were recorded with Presentation (Neurobehavioral Systems, v21.1, 2019). Online sessions—including the active CBM-I and sham training, the ERT, and follow-up assessments—were conducted using a custom-built online platform implemented with Java Server Pages and JavaScript on a secure server at Ruhr University Bochum (adapted by FW from the platform used in previous studies^6^).

Continuous EEG was recorded using a BrainVision actiCAP system (Brain Products GmbH, Munich, Germany) and BrainVision Recorder software (v1.23.0003, Brain Products GmbH, Munich, Germany), sampled at 1,000 Hz with an online notch filter (50Hz), from 32 scalp locations, according to the International 10/20 system.

Periphysiological parameters, i.e., HR and HRV, were recorded during the entire course of the study, see flow chart for the corresponding time points per session. To assess HR and HRV, an Einthoven Lead-II ECG (sampling rate 1000 Hz; digitization 16 bit) was recorded using disposable Ag/AgCl electrodes attached to the right clavicle and the lowest rib on the left side within the axillary line.

Endocrine stress response, i.e., cortisol levels and sAA were measured during the first and second lab session, at different timepoints, as described earlier. Participants received a detailed briefing in advance with all the information they needed about how to prepare for the testing. They were asked not to participate on an empty stomach on the day of the study, but were advised not to eat, chew gum, or drink anything except water (especially stimulants such as coffee, black tea, etc.) for 1-2 hours before the study, not to brush their teeth, and to refrain from consuming nicotine-containing products. Salivary samples were obtained using Salivette sampling devices (Sarstedt, Nümbrecht, Germany). Saliva samples were stored in a freezer (−20 °C). They were analyzed at the biochemical lab of the departments of cognitive psychology and genetic psychology at Ruhr University Bochum.

## **Data Preprocessing**

For all questionnaires, scoring guidelines were followed in accordance with the original questionnaire publication. Both tasks, ERT and SRT, were scored in accordance with the pre-registered protocol (described above in the ‘Secondary Outcomes’ subsection).

N400 amplitudes: For ERP analysis, the event-locked EEG data were analyzed using EEGLab^7^ (version 2024.2) and ERPLab plugin^8^ (version 9.10) running on MATLAB (MathWorks; version R2024b) routines. Offline, data was downsampled to 250 Hz. A highpass filter of 0.1 Hz was applied at the beginning of preprocessing and a lowpass filter of 30 Hz was applied at the end. Raw EEG data was rereferenced offline to the digital average of the 32 EEG electrodes using the fullRankAveRef plugin (Miyakoshi, 2017). EEG deflections resulting from eye blinks and muscle movements were corrected using independent component analysis (ICA) and the ICLabel plugin^9^. Any remaining artifacts that exceeded ±100 μV in amplitude were rejected within a window size of 200 ms and a window step of 50 ms. Continuous, clean data was epoched into 1,000 ms window relative to the onset of the target word, with a baseline correction of 200 ms before target onset. N400 amplitudes were measured in the 300-450 ms time window post-stimulus onset (i.e., the target word) at the Cz, C3, C4, Pz, P3, and P4 electrode sites, in line with previous literature on the N400 and IB^10,11^ as well as reviews on the N400^12^.

For ERP analysis, EEG recordings were excluded from analyses due to extreme noise based on two criteria: (1) excessive channel loss, if more than 4 of the 32 EEG electrodes were automatically rejected (i.e., > 12.5 % of channels flagged by the clean_rawdata plugin^13^)^[[1]](#footnote-1)^; (2) excessive epoch rejection, if after the application of ICA and ICLabel, more than 25% of epochs were automatically rejected due to noise^14^, based on the ±100 μV criterion mentioned above. Out of a total of 176 sessions (88 participants at 2 time points), these criteria lead to the rejection of 54 sessions, leaving a total of 122 sessions to analyze (75 participants with at least 1 session). In the remaining sessions, on average, 6.7% of bins were excluded (6.3% from T1 and 7.1% from T2) due to excessive noise (±100 μV criterion, as mentioned above).

For FAA analysis, continuous resting‐state EEG recordings were first downsampled to 256 Hz using EEGLAB (v2024.2) in MATLAB R2024b. After re‐referencing to the average of all 32 channels with the fullRankAveRef plugin, a highpass filter of 1 Hz and a lowpass filter of 30 Hz were applied. ICA was then performed, and ICLabel was used to flag and remove components of eye or muscle‐related artifacts. Cleaned continuous data were segmented into 60 s epochs around eyes‐open and eyes‐closed markers; each epoch was further divided into non‐overlapping 2 s segments. A combined artifact‐detection routine flagged epochs in which any channel violated one of six criteria—consecutive‐sample step > 50 μV, sample outside ±75 μV, overall range > 100 μV, 200 ms sliding window peak-to-peak > 100 μV, 100 ms window peak-to-peak < 0.5 μV (flatline), or instantaneous slope > 40 μV/ms—and rejected any epoch with > 10 % of channels flagged^15–17^. Then overlapping 2 s windows (50 % overlap) were created. Each 2 s segment was tapered with a Hanning window and subjected to Fast Fourier Transform (FFT). Alpha power (8–13 Hz) was extracted at F3 and F4, log‐transformed, and asymmetry computed as ln(F4) − ln(F3). Mean asymmetry scores were then calculated separately for eyes‐open and eyes‐closed conditions.

Resting state recordings were excluded from analyses due to extreme noise based on the same two criteria as the ERP recordings. This led to the exclusion of 5 sessions. In the remaining sessions, on average, 2% of segments were excluded due to excessive noise, based on the criteria mentioned above^[[2]](#footnote-2)^.

Electrocardiographic activity was continuously recorded in Einthoven Lead II at a sampling rate of 1000 Hz with 16-bit resolution. Ag/AgCl electrodes were positioned at the participant’s lowest left rib (axillary line) and on both clavicles. Data acquisition was performed with a BrainAmp ExG amplifier (Brain Products GmbH, Gilching, Germany) in combination with BrainVision Recorder software (Version 1.23.0003; Brain Products GmbH, Gilching, Germany), applying an online 50 Hz notch filter. For offline preprocessing, ECG signals were band-pass filtered between 5 and 35 Hz (24 dB/oct), and ectopic beats as well as artifacts were detected and corrected. After detrending, interbeat interval series were tapered with a Hamming window and subjected to fast Fourier transformation to derive frequency-domain indices. With Kubios HRV software (Version 2.1), HRV parameters were calculated^18^. For statistical analyses, mean HR and HRV values were extracted from defined 5-minute segments. HR was analyzed in beats per minute, while HRV was represented by the natural log-transformed root mean square of successive interbeat interval differences (ln RMSSD) and the natural log-transformed high-frequency power (ln HF-Power).

Salivary cortisol concentrations were extracted from the samples using a time-resolved fluorescence immunoassay (IBL, Hamburg, Germany) at the Genetic Psychology Lab of Ruhr University Bochum and are reported in nanomole per liter (nmol/l). Intra- and inter-assay coefficients of variations were below 10.00%. sAA concentrations were assessed using a colorimetric test with 2-chloro-4-nitrophenyl-α-maltro-triosoide (CNP-G3) as the substrate reagent^19^, with inter- and intravariation coefficients below 10%.

Outlier handling was performed for mean HR and each HRV and saliva measure (HRV: natural log transformed root mean square of successive inter-beat-interval differences (RMSSD, ln) and natural log transformed power in high-frequency range (HF-Power, ln)); saliva: sAA and cortisol), using the ‘rstatix’ package^20^ and in line with previous literature and recommendations^21^. This procedure ensured that extreme or unreliable physiological values did not bias subsequent analyses, while preserving valid measurements at both the singular timepoint level (i.e., for each participant at each time, T1 or T2) and variable level. See Table S1 for full details of missing data (either due to technical issues or due exclusion during preprocessing (i.e., outliers or too much noise)).

**Table S1**

*Summary of Missing/Excluded Data*

| Measure |  | Baseline (T1) | Training (T1.5) | Post-training (T2) | Follow-up (T3) | Total present *N* / measurements | % Missing |
| --- | --- | --- | --- | --- | --- | --- | --- |
| LSAS | Missing | 0 | NA | 0 | 7 |  |  |
|  | *N* | 88 |  | 88 | 81 | 257 | 2.65% |
| SPAI | Missing | 0 | NA | 0 | 6 |  |  |
|  | *N* | 88 |  | 88 | 82 | 258 | 2.27% |
| DASS | Missing | 0 | NA | 1 | 6 |  |  |
|  | *N* | 88 |  | 87 | 82 | 257 | 2.65% |
| BFNE | Missing | 1 | NA | 1 | 3 |  |  |
|  | *N* | 87 |  | 87 | 85 | 259 | 1.89% |
| ERT | Missing | 0 | 5 | 0 | 3 |  |  |
|  | *N* | 88 | 83 | 88 | 85 | 344 | 2.27% |
| EQ | Missing | 0 | NA | NA | NA |  |  |
|  | *N* | 88 |  |  |  | 88 | 0.00% |
| NEQ | Missing | NA | NA | NA | 6 |  |  |
|  | N |  |  |  | 82 | 82 | 6.82% |
| Anagram Task | Missing | NA | NA | 0 | NA |  |  |
|  | *N* |  |  | 88 |  | 88 | 0.00% |
| Feedback | Missing | NA | NA | NA | 8 |  |  |
|  | *N* |  |  |  | 80 | 80 | 9.09% |
| Negative Affect | Missing | 2 | NA | 1 | NA |  |  |
|  | *N* | 262 |  | 527 |  | 789 | 0.38% |
| SRT | Missing | 0 | NA | 0 | NA |  |  |
|  | *N* | 88 |  | 88 |  | 176 | 0.00% |
| N400 | Missing | 25 (excluded during pre-processing) | NA | 29 (excluded during pre-processing) | NA |  |  |
|  | *N* | 63 |  | 59 |  | 122 | 30.68% |
| FAA | Missing | 4 (excluded during preprocessing) | NA | 1 (excluded during preprocessing) | NA |  |  |
|  | *N* | 84 |  | 87 |  | 171 | 2.84% |
| Saliva - Cortisol | Missing | 14 (outliers) | NA | 18 (outliers) | NA |  |  |
|  | *N* | 74 |  | 334 |  | 408 | 7.27% |
| Saliva – sAA | Missing | 6 (outliers) | NA | 18 (outliers) | NA |  |  |
|  | *N* | 82 |  | 334 |  | 416 | 5.45% |
| ECG - mean HR | Missing | 7 (technical issues / noise) + 2 outliers = 9 | NA | 30 (technical issues / noise) | NA |  |  |
|  | *N* | 167 |  | 498 |  | 665 | 5.54% |
| ECG - RMSSD | Missing | 7 (technical issues / noise) + 2 outliers = 9 | NA | 30 (technical issues / noise) + 31 outliers = 61 | NA |  |  |
|  | *N* | 167 |  | 467 |  | 634 | 9.94% |
| ECG - HF-Power | Missing | 7 (technical issues / noise) + 11 outliers = 18 | NA | 30 (technical issues / noise) + 47 outliers = 77 | NA |  |  |
|  | *N* | 158 |  | 451 |  | 609 | 13.49% |

*Note.* LSAS = Liebowitz Social Anxiety Scale, SPAI = Social Phobia and Anxiety Inventory, BFNE = Brief Fear of Negative Evaluation, DASS = Depression, Anxiety and Stress Scale, ERT = Encoding Recognition Task, EQ = Expectancy Questionnaire, NEQ = Negative Effects Questionnaires, SRT = Scenario Rating Task, sAA = salivary alpha-amylase, ECG = electrocardiogram, HR = heart rate, HF-Power = high frequency power.

## **Analyses**

Similarly to the main analyses, all supplementary analyses were run using RStudio.

Three scales have been applied only once (EQ at T1, NEQ and feedback at T3) and contain several independent items (i.e., not one total score). For these measures, an independent samples t-test was conducted to examine group differences. All p-values have been adjusted for multiple comparisons using the Benjamini–Hochberg FDR correction.

### ***Negative State Affect***

Positive items in the negative affect scale were reverse scored. The total score was an average score of all items, where higher scores indicated more negative state affect.

Specific planned contrasts were added to the LMM analysis, examining state affect in relation to the stressor (anagram) at T2. These contrasts include: state affect pre- vs. post-anagram (measurement 4 vs. measurement 5) and pre-anagram vs. recovery (measurement 3 vs. measurement 6; i.e., return to baseline). For each contrast, the difference between the two groups was also examined.

### ***Expectancy Questionnaire (EQ)***

For the EQ, each item is averaged on its own scale.

### ***Negative Effects Questionnaire (NEQ)***

Three scores are calculated: first, how many negative incidents occurred (answered as ‘yes’ in response to ‘Did you experience this incident?’). Second, for items which were answered as ‘yes’ – the mean rating of how negatively this incident has affected the participants. Third, for items that were answered as ‘yes’ - how many times the participants indicated that this incident was caused due to participation in the study (and not due to a different reason).

### ***Anagram Task Accuracy***

For each group, accuracy in the anagram task was measured in precents.

### ***Feedback***

Each item in the feedback questionnaire was treated separately. For each item, a group comparison was conducted.

### **Adverse Events Monitoring**

The following Adverse Events were pre-specified: worsening of SAD symptoms (i.e., reliable deterioration, indicated by a significant increase in the LSAS-SR score from baseline to follow-up using the Reliable Change Index^22^, using the value 25.97 (i.e., 26 or above) calculated in a German sample of patients with SAD^23^; acute suicidality or self-harm communicated to or detected by the researchers during the trial. Further potential adverse events were be monitored via the NEQ. Potential relatedness of Adverse Events to the study procedures will be assessed via participant feedback and the NEQ, and by comparing frequencies across trial arms.

### **Reliability**

Reliability was measured for all questionnaires (LSAS, DASS-21, BFNE, SPAI-G) and the two interpretation bias tasks (ERT and SRT). Internal consistency (Cronbach’s α) was calculated for questionnaire measures, reflecting the coherence of items within each scale^24^. For behavioral tasks, split-half reliability (Spearman–Brown corrected) was used, as trials within tasks are not psychometric “items” but repeated measurements, making α inappropriate^25,26^. Cronbach’s alpha was calculated using the ‘psych’ package^27^ on RStudio (same R and RStudio versions as the main analyses).

# **Results**

## **Linear Mixed Models (LMMs)**

Full LMM results for each outcome’s main effects and interaction effects can be found in Table S2.

**Table S2**

LLM Results

| Variable | Effect | Value | *SE* | *df* | *t*-value | *p*-value |
| --- | --- | --- | --- | --- | --- | --- |
| Liebowitz Social Anxiety Scale (LSAS) | (Intercept) | 54.318 | 3.732 | 165 | 14.553 | 0 |
|  | time2 | -5.432 | 2 | 165 | -2.717 | 0.007 |
|  | time3 | -5.891 | 2.605 | 165 | -2.261 | 0.025 |
|  | groupSham | 5.523 | 5.278 | 86 | 1.046 | 0.298 |
|  | time2:groupSham | 1.568 | 2.828 | 165 | 0.555 | 0.58 |
|  | time3:groupSham | -1.351 | 3.727 | 165 | -0.363 | 0.717 |
| Depression, Stress and Anxiety (DASS-21) | (Intercept) | 18.068 | 1.64 | 165 | 11.014 | 0 |
|  | time2 | -1.291 | 1.197 | 165 | -1.079 | 0.282 |
|  | time3 | -2.117 | 1.544 | 165 | -1.371 | 0.172 |
|  | groupSham | -0.523 | 2.32 | 86 | -0.225 | 0.822 |
|  | time2:groupSham | -1.323 | 1.687 | 165 | -0.784 | 0.434 |
|  | time3:groupSham | 0.449 | 2.199 | 165 | 0.204 | 0.839 |
| Brief Fear of Negative Evaluation (BFNE) | (Intercept) | 43.672 | 1.463 | 167 | 29.847 | 0 |
|  | time2 | -1.831 | 0.804 | 167 | -2.278 | 0.024 |
|  | time3 | -3.49 | 1.044 | 167 | -3.342 | 0.001 |
|  | groupSham | -1.399 | 2.066 | 86 | -0.677 | 0.5 |
|  | time2:groupSham | 1.808 | 1.137 | 167 | 1.59 | 0.114 |
|  | time3:groupSham | 3.97 | 1.491 | 167 | 2.663 | 0.009 |
| Social Phobia and Anxiety Inventory (German; SPAI-G) | (Intercept) | 68.333 | 2.993 | 166 | 22.835 | 0 |
|  | time2 | -3.672 | 1.424 | 166 | -2.578 | 0.011 |
|  | time3 | -7.256 | 2.222 | 166 | -3.266 | 0.001 |
|  | groupSham | -0.91 | 4.232 | 86 | -0.215 | 0.83 |
|  | time2:groupSham | 2.532 | 2.014 | 166 | 1.257 | 0.211 |
|  | time3:groupSham | 3.685 | 3.142 | 166 | 1.173 | 0.243 |
| Scenario Rating Task (SRT) | (Intercept) | 3.939 | 0.05 | 602 | 78.557 | 0 |
|  | time2 | 0.017 | 0.07 | 602 | 0.243 | 0.808 |
|  | groupSham | -0.04 | 0.071 | 86 | -0.561 | 0.576 |
|  | valenceSocial | -1.49 | 0.07 | 602 | -21.265 | 0 |
|  | congruenceIncongruent | -2.91 | 0.07 | 602 | -41.544 | 0 |
|  | time2:groupSham | 0.028 | 0.099 | 602 | 0.287 | 0.774 |
|  | time2:valenceSocial | 0.309 | 0.099 | 602 | 3.116 | 0.002 |
|  | groupSham:valenceSocial | -0.027 | 0.099 | 602 | -0.277 | 0.782 |
|  | time2:congruenceIncongruent | -0.034 | 0.099 | 602 | -0.344 | 0.731 |
|  | groupSham:congruenceIncongruent | 0.038 | 0.099 | 602 | 0.382 | 0.702 |
|  | valenceSocial:congruenceIncongruent | 2.73 | 0.099 | 602 | 27.56 | 0 |
|  | time2:groupSham:valenceSocial | -0.122 | 0.14 | 602 | -0.872 | 0.384 |
|  | time2:groupSham:congruenceIncongruent | -0.012 | 0.14 | 602 | -0.088 | 0.93 |
|  | time2:valenceSocial:congruenceIncongruent | -0.605 | 0.14 | 602 | -4.319 | 0 |
|  | groupSham:valenceSocial:congruenceIncongruent | 0.037 | 0.14 | 602 | 0.264 | 0.792 |
|  | time2:groupSham:valenceSocial:congruenceIncongruent | 0.357 | 0.198 | 602 | 1.802 | 0.072 |
| N400 (Simplified Model) | (Intercept) | 0.734 | 0.138 | 410 | 5.331 | 0 |
|  | congruenceIncongruent | -0.539 | 0.139 | 410 | -3.874 | 0 |
|  | valenceSocial | -0.224 | 0.139 | 410 | -1.613 | 0.108 |
|  | congruenceIncongruent:valenceSocial | 0.44 | 0.197 | 410 | 2.237 | 0.026 |
| N400 (Full Model) | (Intercept) | 1.033 | 0.237 | 399 | 4.363 | 0 |
|  | time2 | -0.039 | 0.33 | 399 | -0.119 | 0.906 |
|  | groupSham | -0.664 | 0.349 | 73 | -1.904 | 0.061 |
|  | valenceSocial | -0.11 | 0.19 | 399 | -0.578 | 0.563 |
|  | congruenceIncongruent | -0.363 | 0.19 | 399 | -1.912 | 0.057 |
|  | time2:groupSham | 0.096 | 0.483 | 399 | 0.198 | 0.843 |
|  | time2:valenceSocial | -0.161 | 0.275 | 399 | -0.585 | 0.559 |
|  | groupSham:valenceSocial | -0.255 | 0.28 | 399 | -0.913 | 0.362 |
|  | time2:congruenceIncongruent | -0.319 | 0.275 | 399 | -1.16 | 0.247 |
|  | groupSham:congruenceIncongruent | -0.257 | 0.28 | 399 | -0.918 | 0.359 |
|  | valenceSocial:congruenceIncongruent | 0.248 | 0.268 | 399 | 0.923 | 0.356 |
|  | time2:groupSham:valenceSocial | 0.359 | 0.402 | 399 | 0.893 | 0.372 |
|  | time2:groupSham:congruenceIncongruent | 0.427 | 0.402 | 399 | 1.062 | 0.289 |
|  | time2:valenceSocial:congruenceIncongruent | 0.089 | 0.388 | 399 | 0.23 | 0.818 |
|  | groupSham:valenceSocial:congruenceIncongruent | 0.464 | 0.395 | 399 | 1.174 | 0.241 |
|  | time2:groupSham:valenceSocial:congruenceIncongruent | -0.295 | 0.568 | 399 | -0.52 | 0.604 |
| Encoding Recognition Task (ERT) | (Intercept) | 2.307 | 0.086 | 586 | 26.853 | 0 |
|  | time1.5 | -0.526 | 0.109 | 586 | -4.81 | 0 |
|  | time2 | -0.452 | 0.108 | 586 | -4.192 | 0 |
|  | time3 | -0.486 | 0.108 | 586 | -4.508 | 0 |
|  | groupSham | -0.15 | 0.121 | 86 | -1.235 | 0.22 |
|  | valencePos | -0.189 | 0.108 | 586 | -1.749 | 0.081 |
|  | time1.5:groupSham | 0.552 | 0.155 | 586 | 3.557 | 0 |
|  | time2:groupSham | 0.47 | 0.153 | 586 | 3.084 | 0.002 |
|  | time3:groupSham | 0.468 | 0.154 | 586 | 3.035 | 0.003 |
|  | time1.5:valencePos | 1.248 | 0.154 | 586 | 8.085 | 0 |
|  | time2:valencePos | 1.018 | 0.153 | 586 | 6.674 | 0 |
|  | time3:valencePos | 1.111 | 0.153 | 586 | 7.284 | 0 |
|  | groupSham:valencePos | 0.059 | 0.153 | 586 | 0.387 | 0.699 |
|  | time1.5:groupSham:valencePos | -1.031 | 0.219 | 586 | -4.707 | 0 |
|  | time2:groupSham:valencePos | -0.695 | 0.216 | 586 | -3.223 | 0.001 |
|  | time3:groupSham:valencePos | -0.906 | 0.218 | 586 | -4.162 | 0 |
| Negative Affect | (Intercept) | 2.272 | 0.076 | 699 | 30.098 | 0 |
|  | time2 | 0.041 | 0.076 | 699 | 0.546 | 0.585 |
|  | groupSham | 0.237 | 0.107 | 86 | 2.225 | 0.029 |
|  | time2:groupSham | 0.053 | 0.107 | 699 | 0.499 | 0.618 |
| Frontal Alpha Asymmetry | (Intercept) | 0.034 | 0.03 | 86 | 1.125 | 0.264 |
|  | time2 | 0.003 | 0.038 | 81 | 0.085 | 0.932 |
|  | groupSham | -0.001 | 0.043 | 86 | -0.024 | 0.981 |
|  | time2:groupSham | 0.025 | 0.054 | 81 | 0.466 | 0.642 |
| Saliva - Cortisol | (Intercept) | 3.845 | 0.378 | 318 | 10.182 | 0 |
|  | time2 | -0.974 | 0.351 | 318 | -2.771 | 0.006 |
|  | groupSham | 0.595 | 0.56 | 86 | 1.062 | 0.291 |
|  | time2:groupSham | -0.004 | 0.524 | 318 | -0.008 | 0.994 |
| Saliva - Alpha-Amylase | (Intercept) | 149.443 | 14.898 | 327 | 10.031 | 0 |
|  | time2 | 14.996 | 11.317 | 327 | 1.325 | 0.186 |
|  | groupSham | -22.222 | 21.279 | 85 | -1.044 | 0.299 |
|  | time2:groupSham | 6.353 | 16.195 | 327 | 0.392 | 0.695 |
| ECG - Mean HR | (Intercept) | 75.265 | 1.768 | 576 | 42.575 | 0 |
|  | time2 | -2.015 | 0.978 | 576 | -2.06 | 0.04 |
|  | groupSham | 0.328 | 2.515 | 85 | 0.13 | 0.897 |
|  | time2:groupSham | -1.462 | 1.4 | 576 | -1.044 | 0.297 |
| ECG - RMSSD (log) | (Intercept) | 3.913 | 0.092 | 543 | 42.463 | 0 |
|  | time2 | 0.009 | 0.039 | 543 | 0.244 | 0.807 |
|  | groupSham | -0.118 | 0.108 | 85 | -1.092 | 0.278 |
|  | EDR | -0.65 | 0.228 | 543 | -2.852 | 0.005 |
|  | time2:groupSham | 0.083 | 0.055 | 543 | 1.518 | 0.13 |
| ECG - HF-Power (log) | (Intercept) | 6.916 | 0.178 | 519 | 38.76 | 0 |
|  | time2 | 0.044 | 0.068 | 519 | 0.65 | 0.516 |
|  | groupSham | -0.134 | 0.199 | 84 | -0.673 | 0.503 |
|  | EDR | -1.574 | 0.478 | 519 | -3.295 | 0.001 |
|  | time2:groupSham | 0.082 | 0.096 | 519 | 0.851 | 0.395 |

*Note*. Reference levels are: time = time1, group = active. For SRT/N400, additional reference levels are: valence = neutral, congruence = congruent. For ERT, additional reference is: valence = negative.
EDR is held as a covariate for HRV measures analyses.

## **Negative State Affect**

Results from the LMM and planned contrasts for negative affect can be seen in Tables S3 and S4, respectively. For an illustration of all measurements, see Figure S1. Generally, results show the both groups had more negative state affect post-anagram compared to pre-anagram, with no group differences. The difference between pre-anagram and recovery was significant only in the sham group, but no significant group differences emerge.

**Table S3**

Negative Affect Results per Time and Group

| Group |  | T1 | T2 |
| --- | --- | --- | --- |
| Active | *N* | *n* = 130 | *n* = 263 |
|  | *M (SD)* | 2.27 (0.55) | 2.31 (0.68) |
|  | Within-group *g* [95% CI] | | -0.02 [-0.45, 0.41] |
| Sham | *N* | *n* = 132 | *n* = 264 |
|  | *M (SD)* | 2.51 (0.58) | 2.60 (0.79) |
|  | Within-group *g* [95% CI] | | -0.09 [-0.52, 0.34] |
| Between-group *g* [95% CI] | | -0.42 [-5.39, -1.42] | -0.06 [-0.48, 0.36] |

*Note.* * *p* < 0.05; ** *p* < 0.01; *** *p* < 0.001. *M* = mean, *SD* = standard deviation.

**Table S4**

*Negative Affect Results for Planned Contrasts (Pre- and Post-Anagram)*

| Group |  | Pre-anagram vs. Post-anagram | Pre-anagram vs. Recovery |
| --- | --- | --- | --- |
| Active | *N* | *n* = 43 |  |
|  | *M (SD)* | 2.26 (0.64), 2.89 (0.75) |  |
|  | Within-group *g* [95% CI] | 0.88*** [5.25, 10.34] |  |
| Sham | *N* | *n* = 44 |  |
|  | *M (SD)* | 2.59 (0.76), 3.16 (0.82) |  |
|  | Within-group *g* [95% CI] | 0.71*** [3.81, 8.46] |  |
| Between-group *g* [95% CI] | | 0.11 [-0.31, 0.52] |  |
| Active | *N* |  | *n* = 44 |
|  | *M (SD)* |  | 2.28 (0.64), 2.18 (0.67) |
|  | Within-group *g* [95% CI] |  | -0.15 [-3.27, 0.62] |
| Sham | *N* |  | *n* = 44 |
|  | *M (SD)* |  | 2.59 (0.76), 2.36 (0.75) |
|  | Within-group *g* [95% CI] |  | -0.30** [-5.45, -1.34] |
| Between-group *g* [95% CI] | |  | 0.28 [-0.13, 0.70] |

*Note.* * *p* < 0.05; ** *p* < 0.01; *** *p* < 0.001.

**Figure S1**

*Negative State Affect by Time and Measurement*


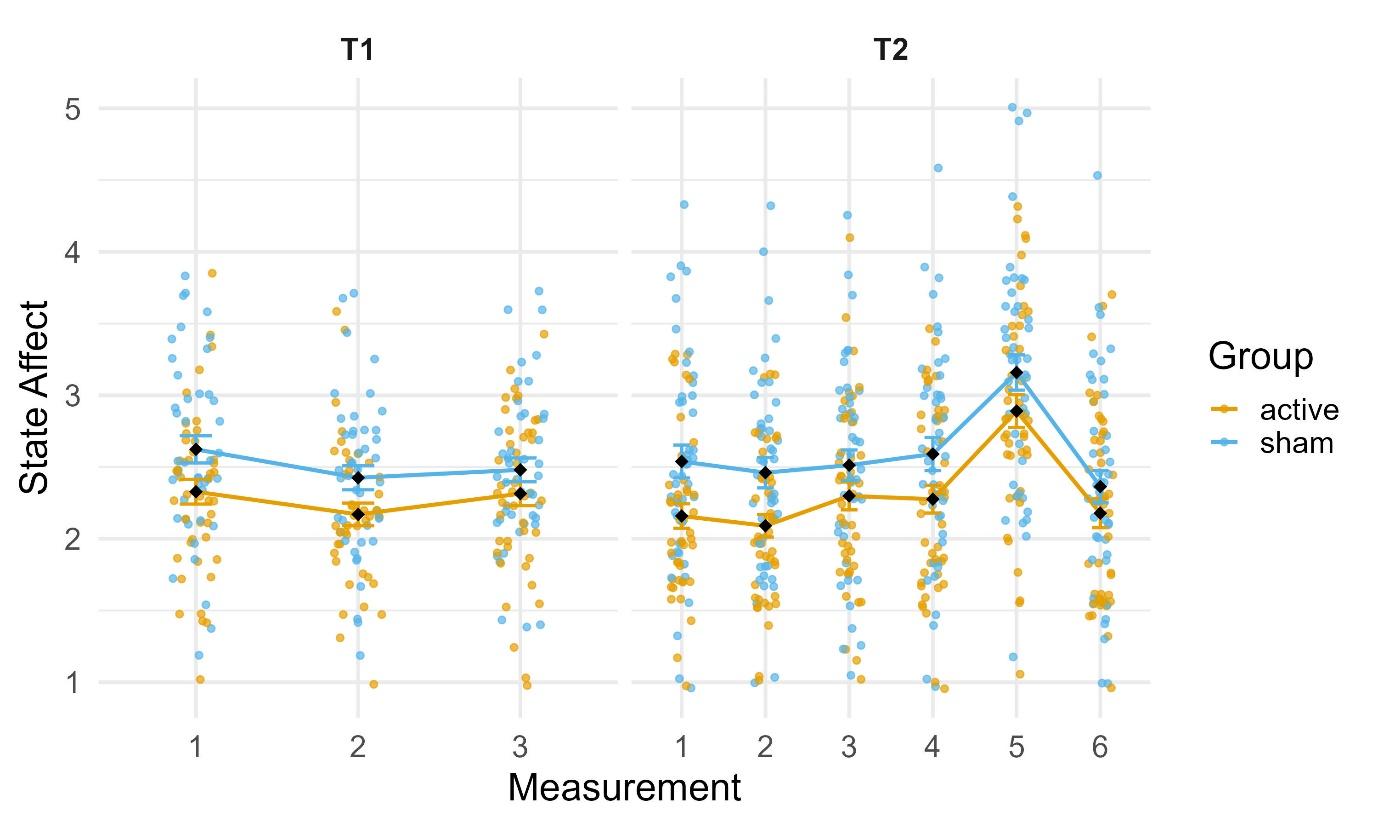


*Note.* Measurements: (1) at baseline, (2) after EEG/ECG preparations, (3) after answering questionnaires, (4) pre-anagram task, (5) post-anagram task, and (6) recovery. The figure shows the mean (in black) and standard error (horizontal lines above and below the mean).

## **Expectancy Questionnaire (EQ)**

At T1, both groups exhibited similar expectancies across all EQ items. For full results, see Table S5.

## **Negative Effects Questionnaire (NEQ)**

At the T3 follow-up, both groups showed highly similar NEQ profiles. For full details, see Table S5.

## **Anagram Task Accuracy**

Accuracy in the anagram task did not differ between the active and sham groups. For more details, see Table S5.

## **Feedback Questionnaire**

Out of 15 feedback items, ratings of 7 items differed between the two groups. For more details, see Table S5.

**Table S5**

*Results from the Anagram Task, EQ, NEQ and Feedback Questionnaire*

| Measure | Scale | Group |  |  |
| --- | --- | --- | --- | --- |
| EQ (1): How much will your impairment due to anxiety symptoms have improved at the end of the computer tasks? | 0-100 | Active | *N* | 44 |
|  |  |  | *M (SD)* | 30.23 (20.85) |
|  |  | Sham | *N* | 44 |
|  |  |  | *M (SD)* | 29.09 (22.60) |
|  |  | Between-group *g* [95% CI] | | 0.05[-0.37, 0.47] |
| EQ (2): How much do you really feel at this point that the computer tasks will help you reduce your impairment due to anxiety symptoms? | 1-9 | Active | *N* | 44 |
|  |  |  | *M (SD)* | 3.41 (1.70) |
|  |  | Sham | *N* | 44 |
|  |  |  | *M (SD)* | 3.59 (1.59) |
|  |  | Between-group *g* [95% CI] | | -0.11[-0.53, 0.31] |
| EQ (3): How much do you really feel your impairment due to anxiety symptoms will have improved? | 0-100 | Active | *N* | 44 |
|  |  |  | *M (SD)* | 24.77 (18.98) |
|  |  | Sham | *N* | 44 |
|  |  |  | *M (SD)* | 24.32 (17.71) |
|  |  | Between-group *g* [95% CI] | | 0.02[-0.39, 0.44] |
| NEQ (1): Incident/effect experienced? | Yes/No - number of 'Yes' responses out of 20 items | Active | *N* | 34 |
|  |  |  | *M (SD)* | 3.76 (2.87) |
|  |  | Sham | *N* | 34 |
|  |  |  | *M (SD)* | 4.35 (2.98) |
|  |  | Between-group *g* [95% CI] | | -0.20[-0.68, 0.28] |
| NEQ (2): If yes, how negatively affected.. | 0 = Not at all to 4 = Extremely | Active | *N* | 34 |
|  |  |  | *M (SD)* | 1.53 (0.58) |
|  |  | Sham | *N* | 34 |
|  |  |  | *M (SD)* | 1.55 (0.74) |
|  |  | Between-group *g* [95% CI] | | -0.03[-0.50, 0.45] |
| NEQ (3): Effects Probably caused by the study or other circumstances | The study (0) vs. other circumstances (1): closer to 0 = closer to study attribution | Active | *N* | 34 |
|  |  |  | *M (SD)* | 0.62 (0.37) |
|  |  | Sham | *N* | 34 |
|  |  |  | *M (SD)* | 0.44 (0.38) |
|  |  | Between-group *g* [95% CI] | | 0.46[-0.02, 0.94] |
| Feedback (1): To what extent do you think the computer task you did made your symptoms worse or better? | -10 (much better) - +10 (not better at all) | Active | *N* | 42 |
|  |  |  | *M (SD)* | -2.12 (2.77) |
|  |  | Sham | *N* | 38 |
|  |  |  | *M (SD)* | 0.08 (3.40) |
|  |  | Between-group *g* [95% CI] | | -0.71** [-1.16, -0.25] |
| Feedback (2): How easy did you find the computer task? | 1 (not easy at all) – 9 (very easy) | Active | *N* | 42 |
|  |  |  | *M (SD)* | 7.12 (1.58) |
|  |  | Sham | *N* | 38 |
|  |  |  | *M (SD)* | 7.26 (1.73) |
|  |  | Between-group *g* [95% CI] | | -0.09[-0.53, 0.35] |
| Feedback (3): How helpful did you find the computer task? | 1 (not helpful at all) – 9 (very helpful) | Active | *N* | 42 |
|  |  |  | *M (SD)* | 4.88 (2.09) |
|  |  | Sham | *N* | 38 |
|  |  |  | *M (SD)* | 3.39 (2.15) |
|  |  | Between-group *g* [95% CI] | | 0.70** [0.24, 1.15] |
| Feedback (4): How stressful or burdensome did you find the computer task? | 1 (not stressful at all) – 9 (very stressful) | Active | *N* | 42 |
|  |  |  | *M (SD)* | 2.71 (1.94) |
|  |  | Sham | *N* | 38 |
|  |  |  | *M (SD)* | 2.74 (1.88) |
|  |  | Between-group *g* [95% CI] | | -0.01[-0.45, 0.43] |
| Feedback (5): If a friend had been in a similar situation, how confident would you be that you would recommend the computer task to others? | 1 (not confident at all) – 9 (very confident) | Active | *N* | 42 |
|  |  |  | *M (SD)* | 4.79 (2.56) |
|  |  | Sham | *N* | 38 |
|  |  |  | *M (SD)* | 4.13 (2.95) |
|  |  | Between-group *g* [95% CI] | | 0.24[-0.21, 0.68] |
| Feedback (6): How pleasant did you find the computer task? | 1 (not pleasant at all) – 9 (very pleasant) | Active | *N* | 42 |
|  |  |  | *M (SD)* | 5.86 (2.08) |
|  |  | Sham | *N* | 38 |
|  |  |  | *M (SD)* | 5.21 (1.80) |
|  |  | Between-group *g* [95% CI] | | 0.33[-0.11, 0.77] |
| Feedback (7): How useful did you find the computer task? | 1 (not useful at all) – 9 (very useful) | Active | *N* | 42 |
|  |  |  | *M (SD)* | 4.88 (1.99) |
|  |  | Sham | *N* | 38 |
|  |  |  | *M (SD)* | 3.29 (2.23) |
|  |  | Between-group *g* [95% CI] | | 0.75** [0.29, 1.20] |
| Feedback (8): How easy was it for you to understand what to do during the computer task? | 1 (very difficult) – 9 (very easy) | Active | *N* | 42 |
|  |  |  | *M (SD)* | 7.93 (1.26) |
|  |  | Sham | *N* | 38 |
|  |  |  | *M (SD)* | 7.76 (1.46) |
|  |  | Between-group *g* [95% CI] | | 0.12[-0.32, 0.56] |
| Feedback (9): How did you find the pace of the computer task? | 1 (far too slow) – 9 (far too fast) | Active | *N* | 42 |
|  |  |  | *M (SD)* | 4.67 (1.07) |
|  |  | Sham | *N* | 38 |
|  |  |  | *M (SD)* | 4.76 (1.13) |
|  |  | Between-group *g* [95% CI] | | -0.09[-0.53, 0.35] |
| Feedback (10): How did you find the length of the individual sessions of the computer task? | 1 (far too long) – 9 (far too short) | Active | *N* | 42 |
|  |  |  | *M (SD)* | 4.02 (1.00) |
|  |  | Sham | *N* | 38 |
|  |  |  | *M (SD)* | 3.66 (1.05) |
|  |  | Between-group *g* [95% CI] | | 0.35[-0.09, 0.80] |
| Feedback (11): Do you think that working on the computer task had a positive influence on your mood? | 1 (not at all) – 9 (very much) | Active | *N* | 42 |
|  |  |  | *M (SD)* | 4.21 (2.05) |
|  |  | Sham | *N* | 38 |
|  |  |  | *M (SD)* | 2.66 (2.08) |
|  |  | Between-group *g* [95% CI] | | 0.75** [0.29, 1.20] |
| Feedback (12): Do you think that working on the computer task had a positive influence on your thoughts? | 1 (not at all) – 9 (very much) | Active | *N* | 42 |
|  |  |  | *M (SD)* | 4.93 (2.20) |
|  |  | Sham | *N* | 38 |
|  |  |  | *M (SD)* | 3.18 (2.01) |
|  |  | Between-group *g* [95% CI] | | 0.82*** [0.36, 1.27] |
| Feedback (13): Do you think that working on the computer task had a positive influence on your behavior/ activities? | 1 (not at all) – 9 (very much) | Active | *N* | 42 |
|  |  |  | *M (SD)* | 4.02 (2.24) |
|  |  | Sham | *N* | 38 |
|  |  |  | *M (SD)* | 2.82 (2.01) |
|  |  | Between-group *g* [95% CI] | | 0.56* [0.11, 1.01] |
| Feedback (14): How satisfied are you with the computer task? | 1 (very dissatisfied) – 9 (very satisfied) | Active | *N* | 42 |
|  |  |  | *M (SD)* | 6.02 (1.49) |
|  |  | Sham | *N* | 38 |
|  |  |  | *M (SD)* | 4.97 (1.79) |
|  |  | Between-group *g* [95% CI] | | 0.63* [0.18, 1.08] |
| Feedback (15): If you found yourself in the same situation, how motivated would you be to do the computer task again? | 1 (not at all motivated) – 9 (very motivated) | Active | *N* | 42 |
|  |  |  | *M (SD)* | 5.19 (2.46) |
|  |  | Sham | *N* | 38 |
|  |  |  | *M (SD)* | 4.79 (2.47) |
|  |  | Between-group *g* [95% CI] | | 0.16[-0.28, 0.60] |
| Anagram (ACC) | 0-1 (0%-100%) | Active | *N* | 44 |
|  |  |  | *M (SD)* | 0.30 (0.14) |
|  |  | Sham | *N* | 44 |
|  |  |  | *M (SD)* | 0.30 (0.12) |
|  |  | Between-group *g* [95% CI] | | 0.00[-0.42, 0.42] |

*Note.* * p < 0.05; ** p < 0.01; *** p < 0.001.

## **Adverse Events Monitoring**

Scores on the NEQ are presented in Table S5, indicating low frequency of negative effects and no differences between trial arms. No Serious Adverse Events were recorded. Three Adverse Events (as pre-specified in the trial protocol) were recorded. These were all reliable deterioration, indexed by LSAS differences from T1 to T3. Reliable deterioration was shown by 1 participant in the CBM group and 2 participants in the sham group (range: 26-44), with no difference between groups (W = 2, p = 0.667). These participants’ feedback (question 1) indicated that they did not consider participation in the study to have worsened symptoms, rating it as neutral (0) or beneficial (−1, −3). These Adverse Events were therefore classed as unlikely to be related to the study.

## **ERT**

Table S6 and Figure S2 show full ERT results, including T1.5.

**Table S6**

*ERT Results by Valence*

| Valence | Group |  | T1 | T1.5 | T2 | T3 |
| --- | --- | --- | --- | --- | --- | --- |
| Negative | Active | *N* | *n* = 44 | *n* = 42 | *n* = 44 | *n* = 44 |
|  |  | *M (SD)* | 2.31 (0.56) | 1.77 (0.56) | 1.85 (0.48) | 1.82 (0.57) |
|  |  | Within-group *g* [95% CI] |  | -0.86***  [-7.79, -3.26] | -0.79*** [-7.40, -2.97] | -0.88***  [-8.13, -3.55] |
|  | Sham | *N* | *n* = 44 | *n* = 41 | *n* = 44 | *n* = 41 |
|  |  | *M (SD)* | 2.16 (0.56) | 2.19 (0.57) | 2.17 (0.54) | 2.15 (0.59) |
|  |  | Within-group *g* [95% CI] |  | 0.05 [-1.59, 2.26] | 0.04 [-1.69, 2.16] | -0.05  [-2.27, 1.58] |
|  |  | Between-group *g* [95% CI] | 0.27 [-0.15, 0.68] | -0.73**  [-1.17, -0.29] | -0.62** [-1.04, -0.19] | -0.56*  [-0.99, -0.13] |
| Positive | Active | *N* | *n* = 44 | *n* = 42 | *n* = 44 | *n* = 44 |
|  |  | *M (SD)* | 2.12 (0.58) | 2.83 (0.57) | 2.68 (0.54) | 2.74 (0.63) |
|  |  | Within-group *g* [95% CI] |  | 1.12*** [4.76, 9.72] | 0.92*** [3.79, 8.43] | 0.93*** [3.82, 8.47] |
|  | Sham | *N* | *n* = 44 | *n* = 41 | *n* = 44 | *n* = 41 |
|  |  | *M (SD)* | 2.03 (0.58) | 2.27 (0.57) | 2.37 (0.55) | 2.22 (0.66) |
|  |  | Within-group *g* [95% CI] |  | 0.45** [0.83, 4.88] | 0.71*** [2.50, 6.83] | 0.30  [-0.07, 3.87] |
|  |  | Between-group *g* [95% CI] | 0.16 [-0.26, 0.57] | 0.97*** [0.51, 1.42] | 0.58** [0.15, 1.00] | 0.80*** [0.35, 1.23] |

*Note.* * *p* < 0.05; ** *p* < 0.01; *** *p* < 0.001.

**Figure S2**

*ERT by Valence and Time*


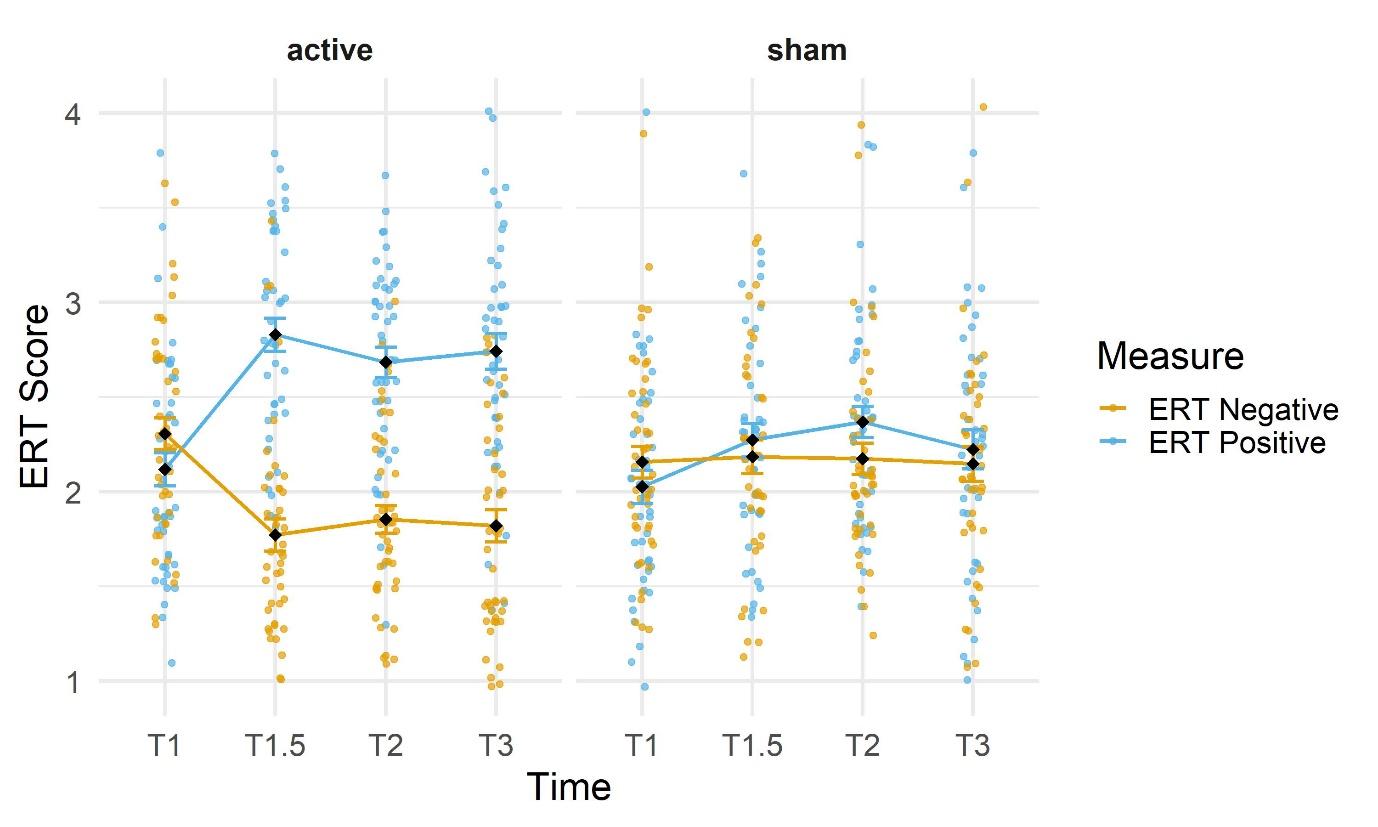


## **SRT – Subjective Ratings**

For a visual representation of the group x time x valence x congruence results of subjective ratings, see Figure S3.

**Figure S3**

*SRT Interaction: Group x Time x Valence x Congruence*


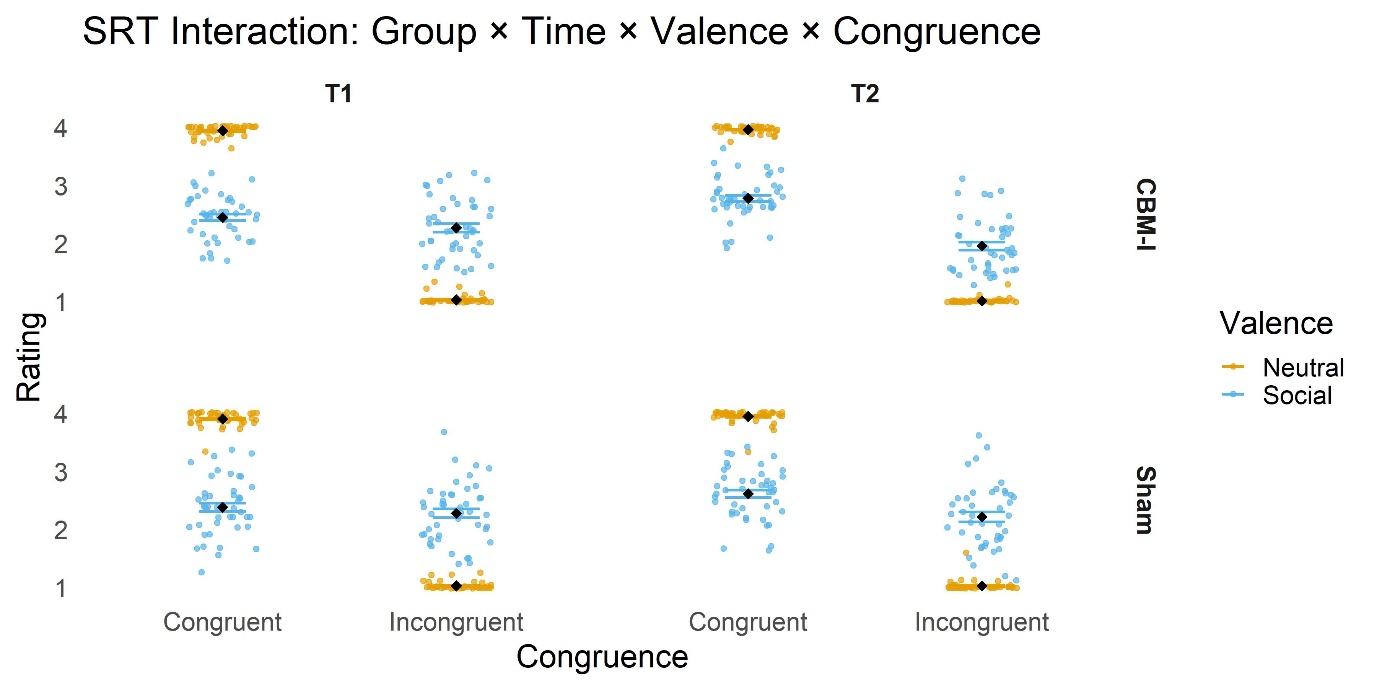
*Note.* For each condition, the figure displays the mean (in black) and standard error (horizontal lines above and below the mean).

## **N400 – Exploratory Analysis**

The main N400 analysis did not show any group or time effects. Thus, as an exploratory analysis, we reanalyzed N400 amplitudes while collapsing these factors and thus included only congruence and valence. For the results of all comparisons, see Table S2. Results of the simplified analysis are displayed in Figure S4.

**Figure S4**

*N400 by Valence × Congruence*


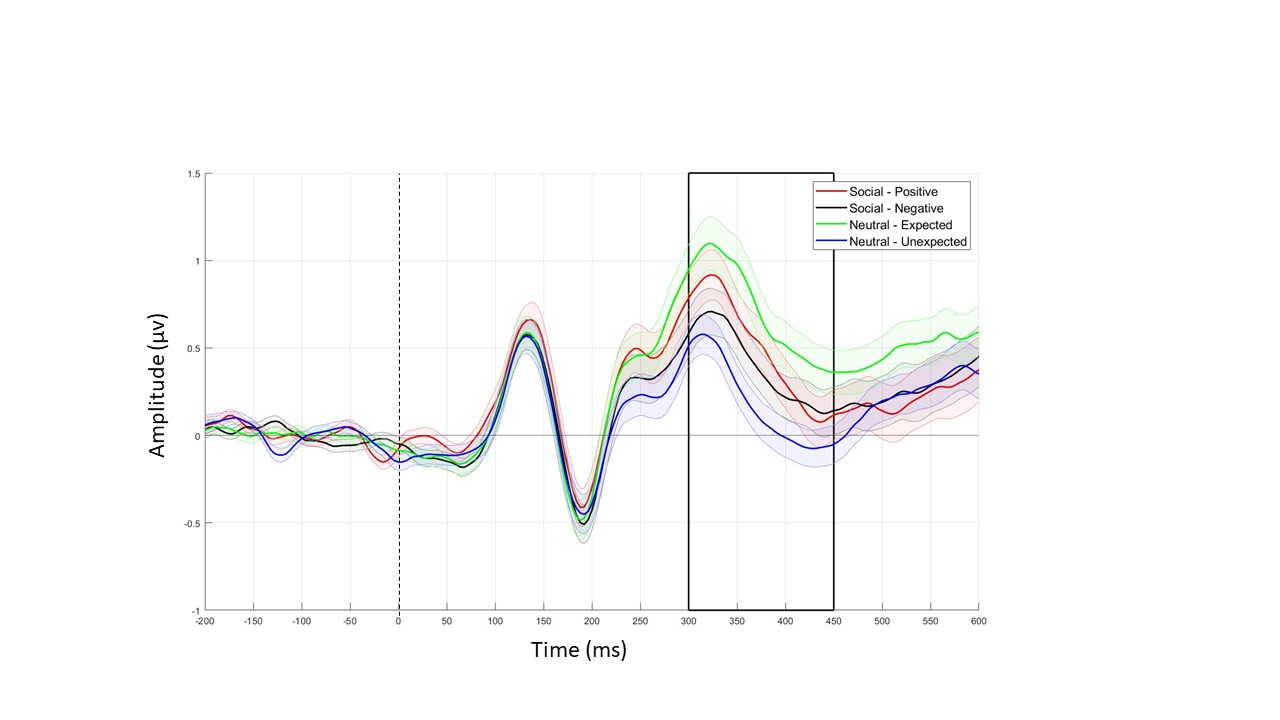


*Note*. Combined plot of N400, representing the mean and standard error for each within-subject condition, for both groups and times (total of 122 sessions across 75 participants). The 0 point on the X axis represents the onset of the final word.

**Table S7**

*N400 Results with Simplified Analysis (Congruence and Valence)*

|  |  | T1 | T2 |
| --- | --- | --- | --- |
| Neutral | *N* | *n* = 63 | *n* = 59 |
|  | Congruent *M (SD)* | 0.73 (1.59) | 0.72 (1.40) |
|  | Incongruent *M (SD)* | 0.25 (1.11) | 0.12 (1.09) |
|  | Within-subject *g* [95% CI] | -0.36** [-4.85, -0.85] | -0.48*** [-5.70, -1.61] |
|  |  |  |  |
| Social | *N* | *n* = 63 | *n* = 59 |
|  | Congruent *M (SD)* | 0.50 (1.50) | 0.50 (1.49) |
|  | Incongruent *M (SD)* | 0.48 (1.50) | 0.32 (1.13) |
|  | Within-subject *g* [95% CI] | -0.02 [-2.12, 1.75] | -0.20 [-3.48, 0.43] |

*Note.* *** p < 0.001. Reported N is out of the initial 88 participants.

Following visual inspection of the results, a brief exploratory analysis was conducted on the same electrode sites as the N400, only in an earlier time window (200-300). The same analysis as the N400 analysis was used. Results showed no interaction between congruence and interaction for the earlier time window, neither for the simplified model (only valence and congruence: *p* = .476), nor for full the model (with time, group, valence and congruence: *p* = .486). This suggests that the interaction between valence and congruence is specific to the N400 window, and that the N400 findings cannot be attributed to an earlier divergence.

## **Mediation (Without Baseline Covariates)**

The mediation model revealed that group assignment significantly predicted negative IB at T2, with participants in the sham condition showing greater negative IB than those in the CBM-I condition (β = 0.22, *SE* = 0.11, *p* = .038). In turn, higher levels of IB significantly predicted higher LSAS scores at T3 (β = 39.03, *SE* = 10.14, *p* < .001). The direct path from group to LSAS scores was not significant (β = -5.33, *SE* = 5.14, *p* = .300), whereas the indirect effect of group on LSAS through IB was significant (β = 8.56, *SE* = 3.89, *p* = .028, 95% CI [1.49, 17.09]). This pattern indicates full mediation, such that the effect of group on social anxiety outcomes at follow-up operated entirely through its impact on IB. Note that all outcomes should be interpreted cautiously: While CFI (.95) and SRMR (.04) suggested adequate fit, RMSEA (.25) and TLI (.71) may be influenced by the limited sample size and low degrees of freedom.

## **HRV Results (Without EDR as Covariate)**

LMM results of both HRV measures without EDR as a covariate are presented in Table S8.

**Table S8**

*HRV LMM Results without EDR as Covariate*

|  | Variable | Value | *SE* | *df* | *t*-value | *p*-value |
| --- | --- | --- | --- | --- | --- | --- |
| ECG - RMSSD (log) | (Intercept) | 3.764 | 0.077 | 545.000 | 49.139 | 0.000 |
|  | time2 | 0.004 | 0.040 | 545.000 | 0.098 | 0.922 |
|  | groupsham | -0.121 | 0.109 | 85.000 | -1.110 | 0.270 |
|  | time2:groupsham | 0.088 | 0.056 | 545.000 | 1.575 | 0.116 |
| ECG - HF-Power (log) | (Intercept) | 6.555 | 0.142 | 521.000 | 46.105 | 0.000 |
|  | time2 | 0.030 | 0.071 | 521.000 | 0.419 | 0.676 |
|  | groupsham | -0.138 | 0.201 | 84.000 | -0.685 | 0.495 |
|  | time2:groupsham | 0.094 | 0.100 | 521.000 | 0.940 | 0.348 |

*Note.* ECG = Electrocardiogram.

## **Reliability**

*Questionnaires*. The LSAS total score, BFNE, and SPAI demonstrated excellent internal consistency across all sessions (Cronbach’s α = .90–.97). The DASS total score showed good to excellent reliability across sessions (α = .89–.94), consistent with established guidelines for questionnaire reliability^24^.

*Tasks*. For the ERT, split-half reliability (Spearman–Brown corrected) was acceptable at all time points and for both positive and negative conditions (α_SB = .77–.86). Split-half reliability for the SRT was high for social sentences (α_SB = .68–.85) and moderate for the neutral sentences (α_SB = .45–.76). The neutral sentences’ poor reliability results are likely due to ceiling and floor effect in the ratings of expected and unexpected outcomes, respectively, as participants consistently rated the unexpected outcomes as unexpected and vice versa, with little variance.

# **References**

1. Turner, S. M., Beidel, D. C., Dancu, C. V. & Stanley, M. A. An empirically derived inventory to measure social fears and anxiety: The Social Phobia and Anxiety Inventory. *Psychological Assessment: A Journal of Consulting and Clinical Psychology* **1**, 35–40 (1989).

2. Fydrich, F. SPAI – Soziale Phobie und Angstinventar. in *Diagnostische Verfahren in der Psychotherapie* (eds Geue, K., Strauß, B. & Brähler, E.) 461–465 (Hogrefe, Göttingen, 2016).

3. Borkovec, T. D. & Mathews, A. M. Treatment of nonphobic anxiety disorders: A comparison of nondirective, cognitive, and coping desensitization therapy. *Journal of Consulting and Clinical Psychology* **56**, 877–884 (1988).

4. Rozental, A. *et al.* The Negative Effects Questionnaire: psychometric properties of an instrument for assessing negative effects in psychological treatments. *Behav. Cogn. Psychother.* **47**, 559–572 (2019).

5. Woud, M. L. *et al.* The Effects of Modifying Dysfunctional Appraisals in Posttraumatic Stress Disorder Using a Form of Cognitive Bias Modification: Results of a Randomized Controlled Trial in an Inpatient Setting. *Psychother Psychosom* **90**, 386–402 (2021).

6. Blackwell, S. E. *et al.* Demonstration of a ‘leapfrog’ randomized controlled trial as a method to accelerate the development and optimization of psychological interventions. *Psychol. Med.* **53**, 6113–6123 (2023).

7. Delorme, A. & Makeig, S. EEGLAB: an open source toolbox for analysis of single-trial EEG dynamics including independent component analysis. *Journal of Neuroscience Methods* **134**, 9–21 (2004).

8. Lopez-Calderon, J. & Luck, S. J. ERPLAB: an open-source toolbox for the analysis of event-related potentials. *Front. Hum. Neurosci.* **8**, (2014).

9. Pion-Tonachini, L., Kreutz-Delgado, K. & Makeig, S. ICLabel: An automated electroencephalographic independent component classifier, dataset, and website. *NeuroImage* **198**, 181–197 (2019).

10. Feng, Y.-C. *et al.* Using event-related potential and behavioural evidence to understand interpretation bias in relation to worry. *Biological Psychology* **148**, 107746 (2019).

11. Feng, Y.-C. *et al.* Impact of imagery-enhanced interpretation training on offline and online interpretations in worry. *Behaviour Research and Therapy* **124**, 103497 (2020).

12. Kutas, M. & Federmeier, K. D. Thirty Years and Counting: Finding Meaning in the N400 Component of the Event-Related Brain Potential (ERP). *Annu. Rev. Psychol.* **62**, 621–647 (2011).

13. Miyakoshi, M. Artifact subspace reconstruction: a candidate for a dream solution for EEG studies, sleep or awake. *SLEEP* **46**, zsad241 (2023).

14. Luck, S. J. *An Introduction to the Event-Related Potential Technique*. (The MIT Press, Cambridge, Massachusetts, 2014).

15. Feldmann, L. *et al.* Resting frontal EEG asymmetry in adolescents with major depression: Impact of disease state and comorbid anxiety disorder. *Clinical Neurophysiology* **129**, 2577–2585 (2018).

16. Heffer, T. & Willoughby, T. A longitudinal study investigating trajectories of sensitivity to threat over time and their association with alpha asymmetry among children and adolescents. *Developmental Cognitive Neuroscience* **46**, 100863 (2020).

17. Monni, A., Collison, K. L., Hill, K. E., Oumeziane, B. A. & Foti, D. The novel frontal alpha asymmetry factor and its association with depression, anxiety, and personality traits. *Psychophysiology* **59**, e14109 (2022).

18. Adolph, D., Zhang, X. C., Teismann, T., Wannemüller, A. & Margraf, J. Respiratory Sinus Arrhythmia—Common and Distinct Mechanisms of Emotional Adjustment in the Depressive and Anxiety Disorders Spectrum? *Psychophysiology* **62**, e70079 (2025).

19. Lorentz, K., Gütschow, B. & Renner, F. Evaluation of a Direct α-Amylase Assay Using 2-Chloro-4-nitrophenyl-α-D-maltotrioside. *cclm* **37**, 1053–1062 (1999).

20. Kassambara, A. rstatix: Pipe-Friendly Framework for Basic Statistical Tests. 0.7.2 https://doi.org/10.32614/CRAN.package.rstatix (2019).

21. Field, A., Miles, J. & Field, Z. *Discovering Statistics Using R*. (SAGE, London, 2012).

22. Jacobson, N. S. & Truax, P. Clinical significance: A statistical approach to defining meaningful change in psychotherapy research. *Journal of Consulting and Clinical Psychology* **59**, 12–19 (1991).

23. Von Glischinski, M. *et al.* Liebowitz Social Anxiety Scale (LSAS): Optimal cut points for remission and response in a German sample. *Clin Psychology and Psychoth* **25**, 465–473 (2018).

24. Cronbach, L. J. Coefficient Alpha and the Internal Structure of Tests. *Psychometrika* **16**, 297–334 (1951).

25. Parsons, S., Kruijt, A.-W. & Fox, E. Psychological Science Needs a Standard Practice of Reporting the Reliability of Cognitive-Behavioral Measurements. *Advances in Methods and Practices in Psychological Science* **2**, 378–395 (2019).

26. Hedge, C., Powell, G. & Sumner, P. The reliability paradox: Why robust cognitive tasks do not produce reliable individual differences. *Behav Res* **50**, 1166–1186 (2018).

27. Revelle, W. psych: Procedures for Psychological, Psychometric, and Personality Research. 2.5.6 https://doi.org/10.32614/CRAN.package.psych (2007).

1. No standardized guidelines were found for the maximum number of electrodes that may be rejected and interpolated within a single recording session. To adopt a conservative approach, we initially applied a threshold of up to three electrodes (i.e., fewer than 10 % of total channels), which led to the exclusion of 15 additional sessions. As analyses conducted without these sessions yielded results identical to those obtained when they were included, we ultimately chose to reintegrate all 15 sessions to preserve statistical power. [↑](#footnote-ref-1)
2. Preprocessing and analysis were conducted both with and without spherical‐spline current‐source‐density (CSD) transformation. Linear mixed-effects models fit to the non-CSD data consistently yielded lower Akaike information criterion (AIC) values than those fit to CSD-transformed data; accordingly, results reported herein are based on the non-CSD preprocessing pipeline. [↑](#footnote-ref-2)
